# Connectome-Based Predictive Modeling of Concurrent and Prospective Substance Use in Adolescence

**DOI:** 10.1101/2025.09.01.673428

**Published:** 2025-09-04

**Authors:** João F. Guassi Moreira, Nicholas Allgaier, Micah E. Johnson, Alexandra Potter, Hugh Garavan, Damien Fair

**Affiliations:** 1 Department of Psychology, University of Wisconsin, Madison; 2 Department of Psychiatry, University of Vermont; 3 David Geffen School of Medicine, University of California, Los Angeles; 4 Institute of Child Development, University of Minnesota, Minneapolis; 5 Masonic Institute for the Developing Brain, University of Minnesota, Minneapolis; 6 Department of Pediatrics, University of Minnesota, Minneapolis

**Keywords:** Substance Use, Adolescence, Connectome-Based Predictive Modeling, Development, rs-fMRI, Connectivity

## Abstract

Understanding the neural mechanisms of adolescent substance use is a critical public health issue, with direct implications for bolstering prevention and treatment strategies. Yet this effort is challenging because substance use is multi-faceted, commonly used brain network features are not optimized to capture both local and global aspects of intrinsic connectivity, and because the facets themselves sensitive to developmental shifts. In this study, we operationalized adolescent substance use along three dimensions—intent, access, and family-developmental history—and trained predictive models of each facet using resting-state connectivity. Trait impulsivity, a known risk factor, was also examined. Using Baseline and 2 Year Follow-Up data from the ABCD Bids Community Collection (ABCC), we found that prediction was more successful at follow-up than baseline. At baseline, predictive accuracy was modest and intent to use substances was the most accurately predicted facet. Prediction accuracies at follow-up were much higher, with access and family-developmental history being better predicted, signaling a developmental shift in the brain–behavior mapping of substance use vulnerabilities. These findings suggest that the neurobiological correlates of substance are dynamic across adolescence, possibly reflecting changing phenotype. More broadly, these results underscore the importance of modeling distinct substance use facets and accounting for developmental timing to understand risk trajectories, while contributing to a growing literature that shows early-developing individual differences are predictive of later outcomes.

## 1. Introduction

The societal costs of substance use are enormous. In the United States alone, the economic burden of alcohol, tobacco, and illicit substances stretches upwards of $700 billion (Rehm et al., 2009; NIDA, 2017). Consequently, a central goal in translational neuroscience is to identify neurobiological markers that can predict substance use and related vulnerabilities. This problem is highly complex. Researchers must determine (i) how to operationalize substance use, (ii) grapple with technical challenges related to selecting specific facets of brain activity to measure and which modeling framework to pair them with, and (iii) build predictive models that account for developmental context in relevant phenotypes. In the current study, we address these challenges by defining substance use as a multidimensional construct, representing intrinsic brain networks using connectome embeddings that capture local and global topology, and evaluating predictive models across multiple developmental timepoints to test their sensitivity to age-related changes in substance use phenotypes.

### 1.1 Background

#### 1.1.1 Operationalizing Substance Use.

The etiology of substance use is myriad, with available literature showing that various factors play a role in shaping use. Foundational genetic variation such as polymorphisms in dopamine-related genes ((Mallard et al., 2016; Ruchkin et al., 2021; Skowronek et al., 2006) have been linked to increased risk. Contextual-environmental factors such as family climate, socioeconomic status, and adverse early experiences appear to dynamically shape substance use tendencies throughout the lifespan (Dick et al., 2007; McGue et al., 2000; Rogers et al., 2022; Silberg et al., 2003). Similarly, sociocultural customs related to social group norms (Fujimoto & Valente, 2012; Hussong, 2002; Lilja et al., 2003) and substance availability (Steen, 2010; Toumbourou et al., 2007) represent additional vulnerabilities to substance use. Notably, these influences on substance use operate through equifinal pathways (Cicchetti & Rogosch, 1996) such that the same behavioral outcomes can be traced back onto diverse developmental histories, suggesting that studying substance use phenotypes and associated vulnerabilities in the collective may be a particularly fruitful method of inquiry.

For these reasons, defining substance use phenotypes in the quest for neurobiological markers is challenging. Even researchers who are interested in use of a single substance, or a particular substance use disorder (e.g., cannabis use disorder), must grapple with the complexity of isolating relevant behavioral phenotypes (such as frequency of use, dosage, delivery method, etc.) and account for the fact that use behaviors and vulnerabilities are often co-occurring across substances (Tomczyk et al., 2016; Vergunst et al., 2022), or that individuals may use substances in such a manner that does not meet clinical diagnostic thresholds but may nevertheless still carry important developmental, health, and societal consequences. Moreover, vulnerability to substance use is oft characterized by presence of transdiagnostic risk factors that are not intrinsically defined in relationship to substance use, but nevertheless evince strong associations with use (e.g., trait impulsivity). Because these vulnerabilities are often of interest in prevention, screening, or treatment efforts, their inclusion is warranted in the search for neurobiological markers.

#### 1.1.2 Technical Hurdles: Measuring and Modeling with Connectivity.

Resting state functional magnetic resonance imaging (rs-fMRI) is a putatively fruitful tool for identifying and understanding neurobiological precursors given its ability to assess core, intrinsic brain networks that are thought to underlie clinically relevant behaviors, cognitions, and motivations (Canario et al., 2021; Petersen et al., 2024; Schettino et al., 2024; Seitzman et al., 2019). The past quarter century has seen a massive surge of interest specifically in using brain network connectivity metrics derived from rs-fMRI as a means for understanding brain-behavior relationships a la predictive modeling (Ooi et al., 2025; Shen et al., 2017; Spisak et al., 2023). For as much optimism as there is in leveraging rs-fMRI to model substance use, there is an accompanying basket of technical hurdles (Uddin et al., 2024; Wu et al., 2023).

One hurdle lies in how functional brain networks are represented. Traditional approaches rely on pairwise Pearson correlations between the functional time series of brain regions. These metrics that are conceptually straightforward but can be noisy and may fail to capture higher-order network structure (Bastos & Schoffelen, 2015; Cutts et al., 2023, 2025; Milisav et al., 2024). This is because brain networks contain both *local* information (connections amongst specific pairs of regions) and *global* topographical information (overall network architecture) (Petersen & Sporns, 2015; Rosenthal et al., 2018). Focusing more one type of information comes at the risk of discarding valuable information about the other. Newer available methods such as connectome embeddings—vector representations learned from network topology by fitting high dimensional computational models—aim to encode both local and global features in a parsimonious form (Rosenthal et al., 2018; Levakov et al., 2018), but have not yet been used extensively in connectome-based predictive modeling.

A second hurdle relates to modeling choices, specifically the use of predictive modeling (e.g., using cross-validation to build a model to predict new, unseen data) versus descriptive approaches (e.g., testing pairwise associations between brain data and phenotypes of interest). While historical attempts at identifying neurobiological markers have favored descriptive models due to their conceptual tractability and ostensible utility for generating causal insights (Yarkoni & Westfall, 2017), they tend to be statistically inefficient and more vulnerable to noise (Spisak et al., 2023). Consequently, brain-behavior phenotypes derived from such approaches often generalize less effectively compared to those identified through predictive modeling methods (Marek et al., 2019, 2022; Spisak et al., 2023; Yarkoni & Westfall, 2017).

#### 1.1.3. Accounting for Developmental Context.

Identifying neurobiological markers of substance use is a developmental science problem. Substance use trajectories often begin within the first two decades of life and indicators of substance use undergo meaningful change as well.

The onset of substance use and related vulnerabilities often emerge in adolescence. This phase in the lifespan is characterized, among other things, by the emergence of heightened risk-taking propensity (Duell et al., 2018; Tervo-Clemmens et al., 2024). While some kinds of risk-taking can potentially facilitate positive developmental outcomes (Do et al., 2017; Duell & Steinberg, 2019), others like substance use can seriously threaten adolescent wellbeing (Cunningham et al., 2018). Substance use in adolescence is increasingly prevalent—approximately 25 to 50% of teens report substance use experimentation during high school (Johnston et al., 2019)—and is highly consequential for usage habits later in life (Centers for Disease Control and Prevention, 2020; Garofoli, 2020). Moreover, vulnerabilities to substance use, such as peer deviance or reduced parental monitoring, also manifest during this time (for instance, as teens become susceptible to peer influences and are granted more autonomy; Blakemore & Mills, 2014; Steinberg & Morris, 2001).

Further compounding this challenge is the fact that substance use and its related vulnerabilities are prone to developmental changes across the lifespan, meaning that relevant indicators be expressed differently in early adolescence compared to mid adolescence compared to young adulthood. That substance use and related vulnerabilities shift during adolescence makes it imperative for predictive models to contend with changing behavioral baselines and developmental trajectories: predicting a phenotype at one age is not equivalent to predicting it years later. Testing whether predictive models generalize across developmental stages is therefore crucial.

### 1.2 Current Study

To address the three challenges enumerated here, we leveraged data from the ABCD Study (Casey et al., 2018), the largest longitudinal neurodevelopmental cohort available.

To grapple with the challenge of operationalizing substance use amidst considerable heterogeneity across many phenotypes, we followed an existing strategy by computing composite measures from a large set of substance use-related variables in the ABCD dataset (Rapuano et al., 2020): (i) *intent* (intentions to use or active use), (ii) *accessibility* (direct or indirect availability of substances), and (iii) *family-developmental history* (parental or familial substance use, including developmental exposure). We also examined trait impulsivity given its role as a strong transdiagnostic risk factor for substance use.

The challenge of balancing local and global structure in networks defined with rs-fMRI data was addressed by deriving network features from functional connectome embeddings (Rosenthal et al., 2018; Levakov et al., 2018). Connectome embeddings are designed to capture both local and global information about network topology in a parsimonious and efficient manner by training a shallow autoencoder neural network (node2vec, Levakov et al., 2018) on a traditional connectivity matrix to produce vector embeddings for each node in the network. By being able to capture information from both global and local network characteristics, embedding-derived features have the potential to enhance predictive modeling outcomes, potentially outperforming traditional metrics. Given the relatively recent advent of connectome embedding models and our novel application of them to predictive modeling in this context, we compared their performance to other connectivity metrics (observed connectivity calculated using a correlation coefficient and correlations implied by pairwise node embedding similarities) in the Supplement (Section S1). Embeddings were employed in a multivariate connectome-based predictive modeling framework to relate functional connectivity metrics to the three substance use facets and trait impulsivity. In tapping connectome-based predictive modeling, we hope to quantify brain-behavior associations in a way that is generative, statistically generalizable, and more robust to noise.

Finally, to deal with the developmentally-relevant nature of substance use, we trained and evaluated connectome-based predictive models on Baseline rs-fMRI data to independently predict each substance use composite and impulsivity at both Baseline and the 2 Year Follow Up. This design allowed us to assess how well brain features at baseline predict concurrent phenotypes and as well as how predictive performance changes when the same facets are measured two years later. We chose to use rs-fMRI connectivity data from Baseline because there is notable interest in understanding early substance use susceptibility (Jackson et al., 2015; Azagba et al., 2015; Green et al., 2024), recent work suggests early neural phenotypes are more consequential for downstream outcomes (Xie et al., 2025), and because no prior work to our knowledge has utilized a full wave of ABCD data to examine adolescent substance use and vulnerabilities using connectome-based predictive modeling or rs-fMRI data (but see Rapuano et al., 2020; Green et al., 2024).

Overall, by integrating multidimensional behavioral phenotypes, complementary brain connectivity representations, and a multi-wave prediction framework, we aim to provide a more complete account of how intrinsic brain networks relate to substance use and its vulnerabilities in adolescence.

## 2. Methods

### 2.1 Participants.

Data for the current project were taken from the ongoing Adolescent Brain and Cognitive Development (ABCD) study (Casey et al., 2018). The ABCD study is a large, multi-site longitudinal study designed to comprehensively study psychological and neurobiological development across the second decade of life, with emphasis placed on understanding links to addiction, substance use, and other varied wellbeing outcomes. Here we give a brief overview of ABCD study details relevant to the current project. In-depth details of study design, recruitment, and full description of measures are available elsewhere (Casey et al., 2018; Garavan et al., 2018). The study recruited over 11,875 children between the ages of 9 to 11 from 21 research sites across the United States and tracked them for several years. A battery of measures was collected from participants, including the rs-fMRI and substance use data used here. Participants were screened based on the quality of resting state fMRI data. We only included from participants who had 10 minutes of usable resting state data following acceptable head motion thresholds (see 2.2 fMRI Data Acquisition & Preprocessing). In total, our sample was comprised of 5,955 participants (see Table 1 for demographics).

### 2.2 fMRI Data Acquisition & Preprocessing.

At baseline, participants completed four resting state scan runs (five minutes each) with eyes open to help ensure at least ten minutes of data below accepted head motion thresholds. All participants were scanned with harmonized protocols across study sites. Further details about fMRI scanning protocols and procedures for the ABCD study have been documented at length elsewhere (Casey et al., 2018). The resting-state fMRI data used here were preprocessed by the ABCD-BIDS Community Collection (ABCC) team (Feczko et al., 2021) with the following additional pre-processing steps conducted by the Developmental Cognition and Neuroimaging Lab at the University of Minnesota. fMRI data were first de-trended and de-meaned over time based on low head-motion volumes with FD values that did not exceed 0.3 mm. Confound regression was then performed to remove several nuisance signals. This meant regressing the resting state timeseries against the mean time series for white matter, cerebrospinal fluid, and global signal, all translational and rotational motion parameters (12 total). The resulting timeseries was then bandpass filtered between 0.008 and 0.09 Hz using a second-order Butterworth filter applied in the forward and backward directions. Data from frames with a FD value greater than 0.3 mm were replaced with interpolated data from remaining frames to avoid re-introducing head motion artifacts (interpolated data discarded from analyses). The human connectome project (HCP) workbench software was used to convert CIFTI dense timeseries into parcellated timeseries following the 333 ROI Gordon parcellation (Gordon et al., 2016). An additional 19 subcortical ROIs were added for a total of 352 parcels. All greyordinate timeseries were then averaged within parcel. Functional connectivity matrices were calculated by taking the Pearson product moment correlation of all pairwise parcel timeseries.

### 2.3 Node Embedding of Functional Connectivity Data.

Node embedding of functional connectivity was conducted using the cepy python package (Levakov et al., 2021). The package uses the node2vec autoencoder algorithm to estimate a vectorized representation of brain nodes that maintains their topological organization. In doing so, the algorithm is able to efficiently balance the capture of both local (edge-level) and global (network-level) information. This represents a potential improvement in multivariate connectome-based predictive modelling, as current approaches that rely on edgewise features may be disproportionately weighing local information.

Embedding the individual nodes (parcels) in a brain network is accomplished by simulating many sequences of random walks across the network (i.e., a sequence of nodes is generated probabilistically according to the ties among all nodes), recording the sequence of nodes along the walk, moving a sliding window over the sequence of nodes to finally predict the central node from its surrounding context nodes. The algorithm is fit by using an autoencoder, a type of fully connected artificial neural network that takes an input, embeds the individual elements into a latent space that preserves a low dimensional representation of the input, and then reconstructs an output.

Connectome embeddings were computed independently for each individual participant, resulting in a unique vector for each parcel. The vector represents the position of that parcel in an *n*-dimensional latent space, where distances between vectors reflect the topological relationships among regions in the functional connectome. Here we specified 30 latent dimensions for our embeddings (see the Supplement, Section S1 for full specification details). As described in Section 2.5.1, the dimension values for all vectors for all parcels were used in our model training and testing procedure. These embeddings theoretically provide an advantage over observed connectivity because of their ability to balance local versus global (edge vs network) level information as well as their parsimonious feature sets (30 latent dimensions × 352 parcels = 10,560 unique features compared to 61,776 unique features with a traditional connectivity matrix).

### 2.4 Operationalizing Substance Use and Trait Impulsivity.

#### 2.4.1 Substance Use.

We took a theory-driven, multidimensional approach to quantifying different facets of substance use in the ABCD dataset. Using a large pool of available substance use related items, we created three composite variables meant to capture distinct facets of substance use at Baseline: *intent, access*, and *family-developmental history*. Composites were computed for the Baseline and 2 Year Follow-Up time points. Items for the composites were identified by a previous study using Baseline data (Rapuano et al., 2020). For composite scores at Baseline, we used the same exact pool of items in the aforementioned study. The 2 Year Follow-Up composites were using the subset of baseline variables that were still collected at that time, as certain baseline variables were excluded from future time points because they were deemed developmentally irrelevant and therefore not collected again. The full lists of variables for each composite at each timepoint are provided in the Supplement (Section S2). The composite for each facet is described in greater detail below. All composite scores were calculated by averaging the relevant variables for each facet.

The *intent* composite reflects prior or ongoing substance use, curiosity about using substances, and desire to use substances. At baseline, this included items from the Lifetime Use Inventory (Lisdahl & Price, 2012), which first asks youths about whether they have *heard* of a substance, and then queries if they have *tried* or *regularly use* each substance via a timeline follow-back assessment (Cervantes et al., 1994). Additional items related to this facet (assessing alcohol, nicotine, and caffeine use) were also available at baseline and thus included. The items about whether youth had heard of each substance were excluded from the 2 Year Follow-Up calculation of this composite because those items were no longer collected.

The *access* composite captures contextual and social factors that facilitate or encourage substance use, such as direct access to substances, lax parental rules or monitoring, and the presence of peers who promote substance use. This composite was based on peer group deviance items referencing substance use, household rules regarding use, and parental risk attitudes toward use. The items for this composite were the same in Baseline and 2 Year Follow-Up calculations since the entire set was collected at both timepoints.

Finally, the *family-developmental history (FDHX)* refers to family history or parental developmental history of substance use, including in utero exposure. For Wave 2, family history items were excluded because these do not change over time; all other items were retained.

To reduce the influence of any outlying cases, scores on each composite at both timepoints were winsorized at 3.5 standard deviations.

#### 2.4.2 Trait Impulsivity.

Trait impulsivity in the ABCD study was measured with an abbreviated youth version of the UPPS-P Impulsive Behavior Scale (Watts et al., 2020). The twenty-item scale taps five theoretically-informed dimensions of impulsivity (lack of perseverance, lack of premeditation, sensation seeking, negative urgency, and positive urgency) using a 1 (agree strongly) to 4 (disagree strongly) Likert scale. Participants’ responses to each statement were summed into a single score, with higher scores indicating more impulsivity. Scores were winsorized using the same criterion as the substance use variables.

### 2.5 Analysis Plan.

#### 2.5.1 Connectome-Based Predictive Modeling of Substance Use and Trait Impulsivity.

We leveraged machine learning and a variant of connectome-based predictive modeling (Shen et al., 2017) to engineer predictive models of substance use facets and trait impulsivity at Baseline and the 2 Year Follow-Up using Baseline rs-fMRI data. This process entails identifying the most relevant network features via pairwise associations with a given outcome variable and then using said features to train and validate a predictive model via machine learning techniques (e.g., cross-validation). Of note, the multivariate variant of connectome-based predictive modeling we employ here is better suited for our purposes given the dependency structure present in the connectivity metrics of interest here (Adkinson et al., 2024; Gao et al., 2019).

Overall, we ran 48 unique specifications of predictive model training and validation by varying the outcome being predicted (4), whether the outcomes were at Baseline or Year 2 Follow-Up (2), data splitting into discovery and validation samples (2), and the type of machine learning model used (3). Below we describe the details for each type of specification, followed by the model training and validation procedure.

##### 2.5.1.1 Outcomes.

The three facets of substance use—intent, access, family-developmental history—in addition to trait impulsivity were specified as dependent variables in modeling.

##### 2.5.1.2 Outcome Timing.

Measures of substance use and trait impulsivity were taken at Baseline and the 2 Year Follow-Up.

##### 2.5.1.3 Data Splitting.

We took advantage of ABCC’s matched_group variable that sorts participants into two arms matched on various demographics including race, ethnicity, sex, and socioeconomic status (Arm 1 *n* = 2929, Arm 2 *n* = 3026; https://nda-abcd-collection-3165.readthedocs.io/latest/recommendations/#2-the-bids-participants-files-and-matched-groups). The model training and validation process leveraged was repeated twice such that each arm was used as both a discovery dataset for feature selection and modeling training, and a confirmation dataset for validation (each across independent iterations).

We chose to use the pre-defined matched group designations from the ABCC collection for model training and validation because they afforded us demographically-matched independent sets. This is in contrast to other recent approaches that perform feature selection and cross-validation in the same sample over many repetitions (Adkinson et al., 2024). Without detracting from other such work, our approach theoretically affords us a relatively better estimate of out-sample predictive ability because the model training and validation sets are completely independent and thus minimize any possibility of test-train leakage.

##### 2.5.1.4 Model Type.

We used three types of models: partial least squares regression (PLSR), ridge regression (RIDGE), and XGBoost (XGB). The former two were implemented in sklearn python package, whereas XGB was implemented with the xgboost python package (XGBRegressor() function). The PLSR and RIDGE models each contained one hyperparameter, the number of components (1 to 10) and λ (l2 penalty term; 1e-6 to 1e6 in 20 linearly spaced intervals). The XGB model specified four hyperparameters: the number of estimators (100 or 200), learning rate (0.01, 0.05, 0.10), max depth (3 or 5), and λ (1, 10, 100). Hyperparameters were rounded to whole integers when necessary (e.g., number of components, max depth).

##### 2.5.1.5 Procedure.

For each outcome variable of substance use, the following procedure was enacted (all implemented with sklearn functions). First, the discovery sample was used for feature selection by chaining the RobustScaler() function with its SelectKBest() function into a pipeline that normalized the predictors and then identified the top 1,000 features evincing the strongest association with the given dependent variable via univariate regression. Next, once all features were selected, nested K-fold cross-validation (K = 5 outer folds) was used to train predictive models for each dependent variable in the discovery dataset. On each iteration of the inner loop, the GridSearchCV() function was used to optimize model hyperparameters by minimizing the mean squared error. The best hyperparameters from the grid search on each inner fold were saved as part of the outer loop partition. Once the procedure had been repeated for all outer folds, the best hyperparameters across all outer folds were averaged and used to estimate the model to the full discovery dataset. The model was then fit to the confirmation dataset and an *R*^*2*^ value was created by correlating model predictions and observed results (Pearson’s *r*) and squaring the result.

While we previously justified our choice to use connectome embeddings in our modeling procedure (e.g., capture of both edge-level and network-level connectivity characteristics while offering greater parsimony than full observed connectivity matrices) rather than traditional connectivity matrices, we also wanted to avoid the possibility that this choice inadvertently reduced model performance. To this end, we compared node embedding performance with traditional connectivity (i.e., observed connectivity), model-implied connectivity (i.e., pairwise cosine similarity among all encoding vectors), and two combined feature sets; these results are provided in the Supplement (Supplementary Tables 2–3).

#### 2.5.2 Non-fMRI Covariates.

Non-fMRI covariates were added to each model as part of the feature selection process described above. An initial set of variables comprising data on biological sex, race, ethnicity and socioeconomic status was assembled from ABCD’s study demographic information. Biological sex (male, female) and ethnicity (Hispanic/Latinx vs not Hispanic/Latinx) were binary coded. Race was quantified as a set of one-hot encoded vectors for all the racial categories assessed in the ABCD study (Alaskan Native, Asian, Black, Chinese, Filipino, Guamanian, Hawaiian Native, Indian, Japanese, Korean, Native American, Samoan, Vietnamese, White, Other Asian, Other Pacific Islander, Other Race). This set also included variables for the ‘don’t know’ and ‘declined’ categories, in addition to a composite variable indicated mixed race status (defined as selecting more than one of the racial categories). Socioeconomic status was included because of its strong relationship to wellbeing across development (Peverill et al., 2021), and was captured by a set of variables comprised of parental education and household income.

The entire set of 23 demographic variables described above was concatenated with the discovery data for each iteration of the aforementioned modeling procedure and feature selection was performed as previously described over the entire set of fMRI and non-fMRI features. This procedure gave us the advantage of adjusting for potential non-brain confounds in a data-driven manner while potentially freeing up additional model degrees of freedom to be used on fMRI predictors.

## 3. Results

### 3.1 Establishing Fidelity of Connectome Embeddings.

Our connectome embeddings faithfully represented functional connectivity as defined by the correlation of rs-fMRI parcel timeseries. Details about this analysis can be accessed in the Supplement (Section S1, Supplementary Figure 1).

### 3.2 Connectome-Based Prediction of Substance Use and Impulsivity.

Full results of our connectome-based approach to modeling different facets of substance use and impulsivity at *Baseline* using five different feature sets are listed in Table 2. In line with the prior literature, we observed modest effect sizes that fell between the range of *r* = [0.019, 0.106], with most of the values typically falling between *r* = (0.05, 0.1). This means that even the best predictive models accounted for at most approximately 1% of the variance in substance use facets.

Results for models predicting *2 Year Follow-Up* substance use and impulsivity are listed in Table 3. Consistent with recent work (Xie et al., 2025), predictive accuracy was generally higher when forecasting later outcomes from baseline rs-fMRI data, particularly for substance use composites. For Intent, Access, and Family-Developmental History, many models exceeded r = 0.10, with some—especially for Family-Developmental History—reaching values between 0.30 and 0.54. In contrast, prediction of Impulsivity remained weak, with all r values below 0.10.

**Figure 1.**
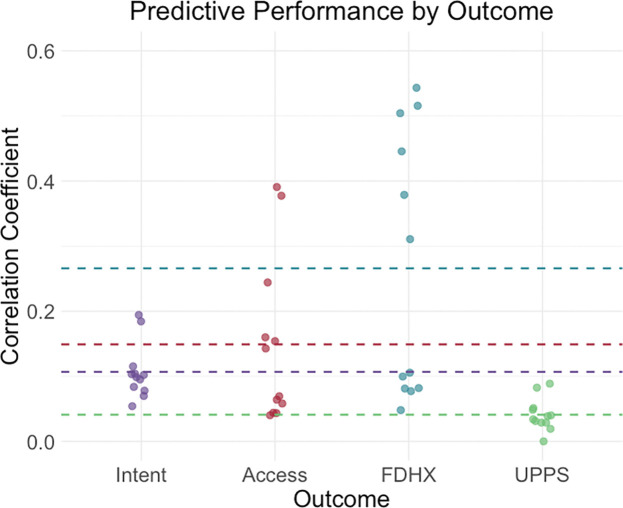
Predictive accuracies were highest for family-developmental history of substance use, collapsing across all other specifications *Note*. ‘FDHX’ refers to family-developmental history; UPPS refers to impulsivity measure with the UPPS-P Impulsive Behavior Scale

**Figure 2.**
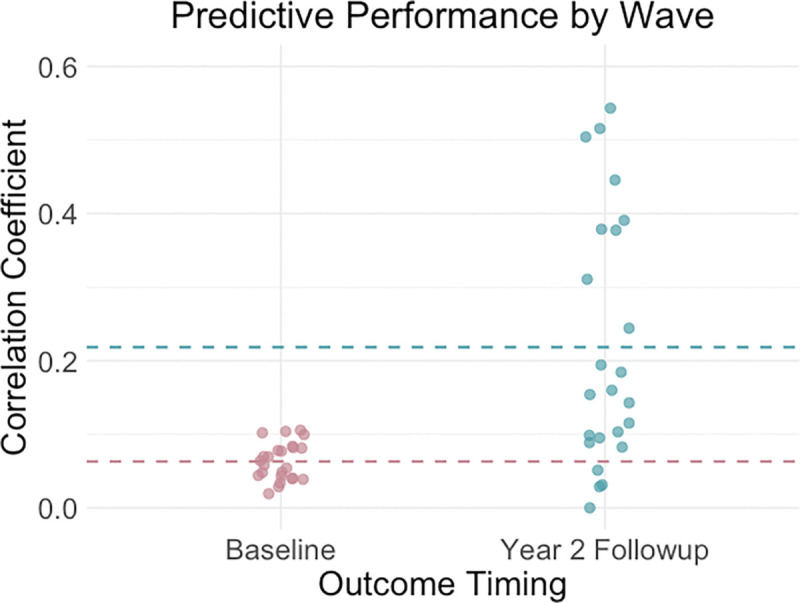
Predictive accuracies were highest for the 2 Year Follow-Up substance use, collapsing across all other specifications. *Note*. Outcome timing refers to timing of when variables used to compute substance use facets were taken.

**Figure 3.**
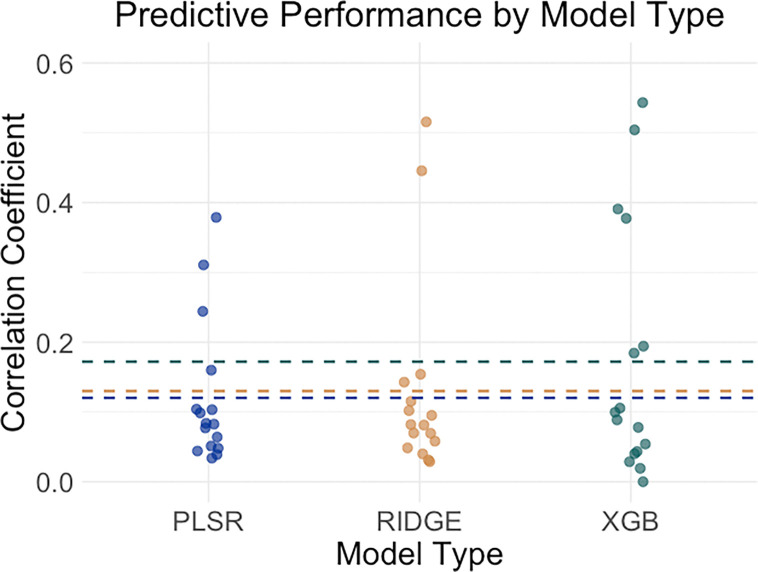
Predictive accuracies were comparable across the type of statistical model used, with a slight edge to XGBoost. *Note*. ‘XGB’, ‘RIDGE’, and ‘PLSR’ refer to models trained and tested with XGBoost, ridge regression, and partial least squares regression respectively. Averages among all correlations in each category is depicted with the hashed lines.

**Figure 4.**
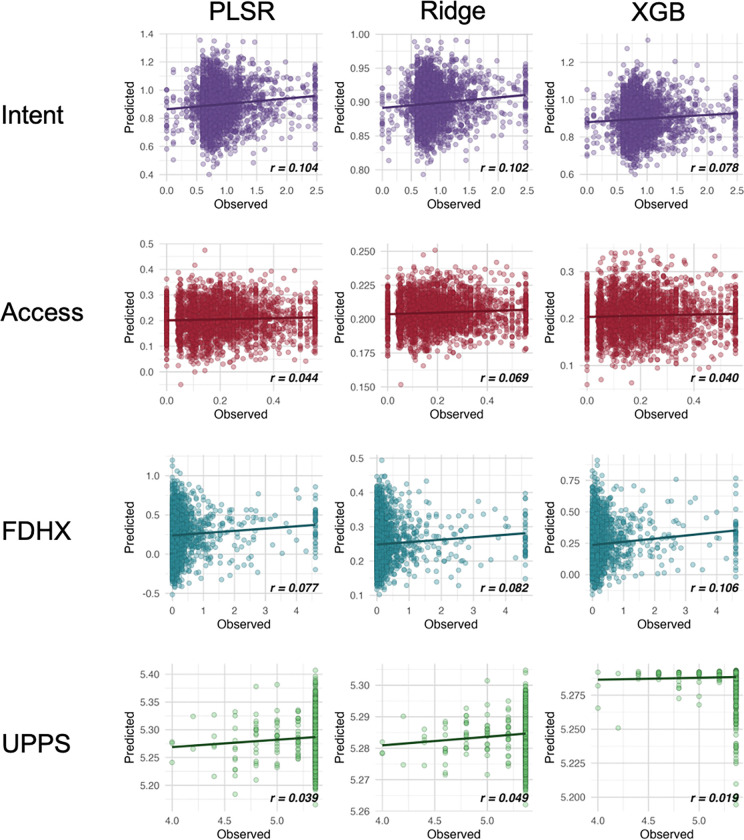
Model accuracy scatter plots by substance use facet at Baseline and model type. *Note*. ‘XGB’, ‘RIDGE’, and ‘PLSR’ refer to models trained and tested with XGBoost, ridge regression, and partial least squares regression respectively. ‘FDHX’ refers to family-developmental history; UPPS refers to impulsivity measure with the UPPS-P Impulsive Behavior Scale. These are results from when Arm 1 was used as the discovery dataset and Arm 2 was used as the confirmation dataset.

**Figure 5.**
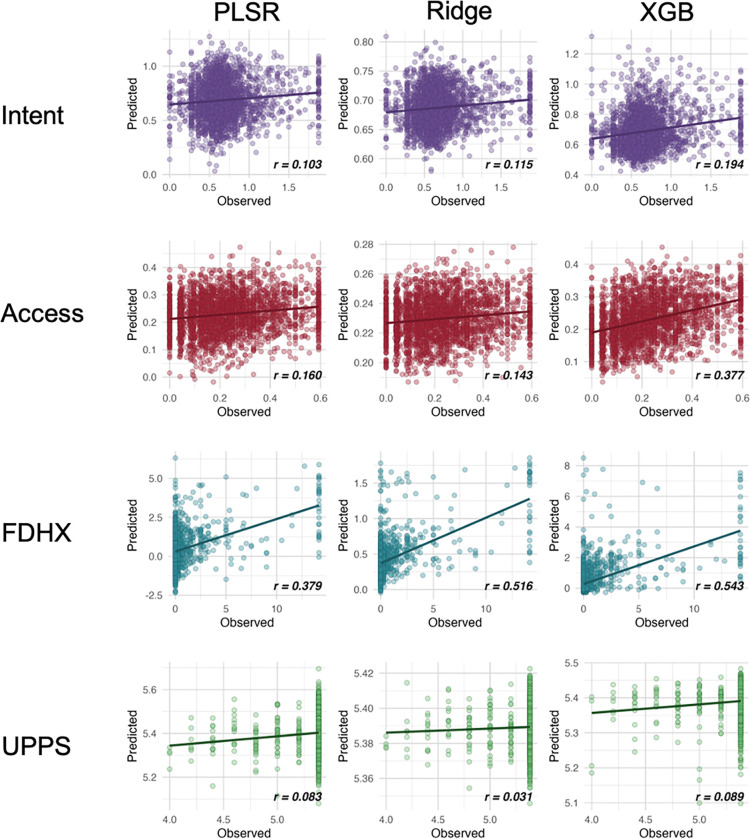
Model accuracy scatter plots by substance use facet at the 2 Year Follow-Up and model type. *Note*. ‘XGB’, ‘RIDGE’, and ‘PLSR’ refer to models trained and tested with XGBoost, ridge regression, and partial least squares regression respectively. ‘FDHX’ refers to family-developmental history; UPPS refers to impulsivity measure with the UPPS-P Impulsive Behavior Scale. These are results from when Arm 1 was used as the discovery dataset and Arm 2 was used as the confirmation dataset.

**Figure 6.**
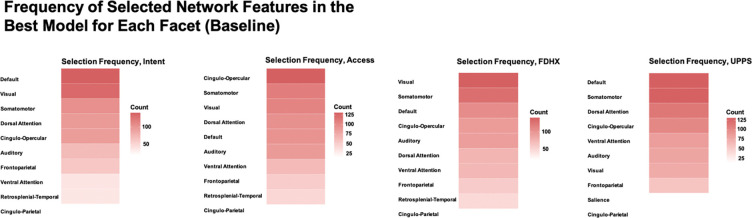
Most important networks, Baseline *Note*. Features come from the best fitting model within each facet for models fit when Arm 2 serve as the confirmation dataset. Network importance was defined by summing the number of features (embedding dimensions) identified in the discover dataset split that belonged to parcels nested within each network.

**Figure 7.**
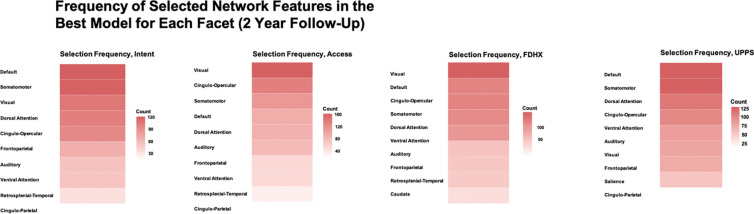
Most important networks, 2 Year Follow-Up *Note*. Features come from the best fitting model within each facet for models fit when Arm 2 serve as the confirmation dataset. Network importance was defined by summing the number of features (embedding dimensions) identified in the discover dataset split that belonged to parcels nested within each network.

We delve into our modeling results in more detail across the following subsections. Figures 1–3 depict modeling accuracy within each of the five modeling specifications; Figures 4–5 plot predicted versus observed scores for every individual modeling instance where matched group arm 1 was used as the confirmation sample (the same plots with arm 2 are included in the Supplement); Figures 6–7 depict the most important model features by whole-brain network (described in detail in Section 3.3).

#### 3.2.1 Predictive Performance by Outcome.

Model performance grouped by outcome (collapsing across all feature specifications and both timepoints) is depicted in Figure 1. Models were most accurate for family developmental history, followed by access, while prediction of impulsivity was consistently poor. These outcome-level differences align with patterns observed across both baseline and follow-up analyses (Tables 2 and 3). Notably, whereas baseline models performed best for intent, follow-up models showed substantially stronger prediction for access and family developmental history. Interpretations should be qualified by the fact that substance use composite definitions differed slightly between waves.

#### 3.2.2 Predictive Performance by Outcome Timing.

Model performance grouped by outcome timing (collapsing across outcomes and feature specifications) is shown in Figure 2. Overall, predictive accuracy was markedly higher at the 2-Year Follow Up compared to baseline, with baseline models clustering near zero and follow-up models frequently exceeding r = 0.2. These differences were driven primarily by the substance use composites, as trait impulsivity was poorly predicted at both timepoints. Importantly, the outcome-specific results in Tables 2 and 3 clarify that this increase in accuracy was especially pronounced for Family-Developmental History and Access, which showed strong improvements at follow-up, whereas Intent was better modeled at baseline.

#### 3.2.3 Predictive Performance by Machine Learning Model Type.

Model performance grouped by machine learning model type (collapsing across outcomes, feature specifications, and timepoints) is shown in Figure 4. Predictive performance was broadly comparable between PLSR and ridge regression, both yielding modest accuracies. By contrast, XGBoost consistently outperformed the linear models, with average correlations higher by approximately 0.06.

#### 3.2.4 Supplementary Comparisons of Predictive Performance by Feature Set.

Supplementary Figure 3 shows a validation analysis comparing node embeddings to alternative connectivity feature sets (observed connectivity, model-implied connectivity, and their combinations). These analyses were conducted to verify that node embeddings perform at least as well as traditional observed connectivity and model-implied connectivity, as well as combinations of these feature sets. Across all five specifications (node embeddings, observed connectivity, model-implied connectivity, observed connectivity + embeddings, and all features combined), predictive performance was broadly comparable. Importantly, feature sets that combined multiple connectivity metrics (e.g., all three metrics together, or observed connectivity plus embeddings) did not confer an accuracy advantage over node embeddings alone, indicating that node embeddings achieve predictive accuracy on par with traditional and combined feature sets, despite being a lower-dimensional representation of network topology at the data level.

#### 3.2.5 Predictive Performance by Sample Split.

We checked to see whether model performance differed based on which matched group served as the confirmation arm. Supplementary Figure 5 shows predictive performance was comparable between sample splits.

### 3.3 Unpacking rs-fMRI Connectivity Features.

Feature importance analyses revealed which large-scale brain networks contributed most strongly to model predictions (Figure 6 outcomes at Baseline; Figure 7 outcomes at the 2 Year Follow-Up). For these analyses, we examined the best-fitting model for each outcome served as the confirmation dataset (Arm 2 depicted for brevity). Embedding dimensions selected in the discovery split were mapped back to their corresponding parcels and further aggregated at the network level.

At Baseline, predictive features were relatively diffuse across networks. Given that baseline models generally showed weak predictive accuracy, this diffuse distribution likely reflects instability in feature selection rather than meaningful network-level contributions. By contrast, at the Year 2 Follow-Up, a clearer and more focused pattern emerged, with the Default Mode, Control, and Salience/Ventral Attention networks contributing disproportionately large numbers of features across the outcomes examined here.

## 4. Discussion

### 4.1 Overview.

The present study sought to probe the underlying neurobiology of adolescent substance use by leveraging predictive modeling and resting state fMRI. In doing so, we aimed to address three key difficulties: (i) how to operationalize substance use, (ii) technical hurdles in rs-fMRI modeling—selecting and representing connectivity features and using predictive modeling rather than descriptive approaches—and (iii) capturing phenotypes that change with development. To that end, we partitioned substance-use measures into three composites, estimated intrinsic connectivity with connectome embeddings, and evaluated predictive performance at both Baseline and the 2 Year Follow-Up. For completeness, we benchmarked embeddings against observed and model-implied connectivity in supplementary analyses. Broadly, we found that predictive accuracy was weak at baseline but improved substantially at the two-year follow-up, with differences emerging across substance use facets.

At baseline, predictive models yielded modest effect sizes, typically accounting for less than 1% of the variance in outcomes, consistent with prior literature. By contrast, predictive accuracy was substantially higher for Wave 2 outcomes, with some models explaining over 10% of the variance. Descriptively, intent was the most predictable facet of substance use at baseline, whereas access and family-developmental history were more accurately predicted in 2 Year Follow Up data. Supplementary analyses showed that node embedding dimensions performed comparably to traditional connectivity metrics, with no consistent advantage for combining feature sets. These findings mark the first connectome-based predictive modeling of substance use to leverage multi-wave ABCD rs-fMRI data, offering new insights into how early brain connectivity forecasts substance use vulnerabilities over time.

### 4.2 Substance Use Intent Was the Most Predictable Facet at Baseline, but Access and Family History Emerged as More Predictive at Follow-Up.

At Baseline, the substance use intent facet—which captures both desire to use substances as well as actual use—was the most accurately predicted by rs-fMRI connectomic data. In contrast, models performed worst when predicting the access facet at this wave. On the one hand, one could argue this pattern was somewhat unexpected given longstanding research emphasizing the central role of contextual factors—such as peer norms and parental monitoring—in shaping substance use behaviors (Fujimoto & Valente, 2012; Hussong, 2002; Lilja et al., 2003; Steen, 2010; Toumbourou et al., 2007), coupled with work from social neuroscience showing that social dynamics, broadly construed, and their correlates are embedded in network connectivity (Hyon et al., 2020, 2022; Rudolph et al., 2018). One possible explanation is that many of the variables comprising the access composite reflect dynamic, situationally dependent factors—such as peer group behavior or household rules—that may lack stable neurobiological correlates at the onset of adolescence or fluctuate too rapidly to be captured reliably by traditional questionnaire measures. On the other hand, one could argue that it is not unexpected to see weak prediction of access at Baseline, because contextual influences on substance use—such as availability, peer access, or parental restrictions—often become more salient only later in adolescence. At ages 9–10, many youths are still under relatively strong parental oversight, have limited autonomy, and may not yet have substantial exposure to peers who use substances. Under such conditions, the brain may have little to “encode” about access in a way that is stable or predictive. Moreover, one could contend that even when these social dynamics do become influential, it may be somewhat far-fetched to expect them to leave robust, readily detectable signatures in intrinsic connectivity measured with rs-fMRI. With that said, however, this initial weakness in predicting access did not persist, as access became among the most predictable facets at the 2 Year Follow Up.

At the 2-Year Follow-Up, the baseline pattern reversed: both access and family-developmental history were predicted more accurately than intent, with access showing especially strong gains in model performance. In the case of family-developmental history, which was only modestly predicted at baseline, the improved prediction at the 2-Year Follow-Up may reflect the accumulating influence of familial and intergenerational risk factors over time. Although one might expect such biologically grounded influences to manifest more strongly in early brain function, our findings suggest that their effects may unfold more gradually—perhaps due to variability in timing, intensity, or cumulative exposure. By contrast, access—which was poorly predicted at baseline—became among the most predictable outcomes at the 2-Year Follow-Up, aligning with developmental shifts in autonomy and peer exposure that make contextual opportunities for use more salient. This pattern highlights the need to consider both developmental timing and latent growth trajectories when interpreting the connectomic signatures of risk.

It is important to note, however, that the composites were not identical across waves: some variables available at baseline were not collected at the 2-Year Follow-Up due to their inappropriateness for older ages (e.g., substance gating items used in the intent composite at Baseline), leading to slight differences in composite composition. While this reflects touches on our efforts to maintain the developmental appropriateness of the composites at each time point, it falls in line with a broader, field-wide methodological challenge for longitudinal work: should measures be kept identical across time points to maximize comparability, or titrated to developmental appropriateness (Telzer et al., 2018)? In the present case, the 2-Year Follow-Up composites reflect the latter approach, as we could have carried over scores from items at Baseline but chose not to. This issue should be kept in mind when interpreting differences in predictive performance between waves (we also discuss this issue more in-depth later in the limitations section). Notably the access composite, which showed stark changes in predictive accuracy between Baseline and Follow-Up, comprised the same items at both timepoints. Similarly, trait impulsivity was poorly predicted at both time points, despite continuity in its constituent items. Both sets of findings suggest that continuities or differences in predictive accuracy are not necessarily solely accounted for by changing the composition of composite variables.

The heterogeneity of our findings serves as an important reminder in the evergreen quest of identifying biomarkers for clinically relevant phenotypes of interest. Different phenotypes can and often do diverge in their neurobiological underpinnings, and these results do not support the idea that substance use behavior, and its related vulnerabilities, are all undergirded by a single common latent neural signature. Instead, they point to the specificity of brain-behavior associations, with different phenotypes exhibiting distinct patterns of predictability. In light of this, we believe that future work may benefit from even more targeted operationalizations of substance use phenotypes of interest where possible.

### 4.3 The Default Mode, Somatomotor, and Cingulo-Opercular networks drove predictive accuracy at Year 2.

At the 2-Year Follow-Up, predictive features were no longer diffusely distributed across the connectome but instead clustered disproportionately within three large-scale systems: the Default Mode, Somatomotor, and Cingulo-Opercular networks. This concentration suggests that as individuals progress into mid-adolescence, the “division of labor” among brain systems relevant to substance use vulnerabilities becomes more focused, with these networks carrying a larger share of predictive signal across outcomes. Importantly, this pattern contrasts with baseline, where weak model performance was accompanied by a diffuse and less interpretable spread of features. Together, these findings indicate that developmental changes are reflected not only in model accuracy but also in the specific network architectures that support prediction.

A closer look at the three networks that dominated predictive features at Year 2 Follow-Up suggests meaningful links to developmental processes relevant for substance use. The default mode network has long been implicated in self-referential thought, social cognition, and affective processing; its prominence here may reflect the increasing role of socioemotional factors, motivational or reward-related drives, and peer-related dynamics in adolescence, which are routinely implicated in substance use initiation (Caouette & Feldstein Ewing, 2017). The cingulo-opercular Network, which supports executive control and salience detection (Gratton et al., 2022), may become more predictive as adolescents’ regulatory capacities are recruited in contexts involving risk, reward, and peer influence. Heightened contributions from this system could reflect the need to detect and manage conflict between long-term goals and immediate opportunities for substance use. The somatomotor network is a less expected contributor (Uddin et al., 2019), but its emergence could signal developmental coupling between sensorimotor processes and substance-related behavior, such as the embodied aspects of substance curiosity or broader integration of motivational and action systems. Although these interpretations are necessarily speculative given the study design, the convergence of these networks underscores that prediction at mid-adolescence reflects not just diffuse connectivity but the coordinated involvement of systems spanning socioemotional, control, and embodied domains.

### 4.4 Connectome Embeddings Show Promising Applications in Connectome-Based Predictive Modeling.

Predictive models trained on node embedding dimensions and model-implied connectivity performed comparably to those based on observed connectivity or combined feature sets. Although these comparisons were conducted as ancillary analyses, the findings are still informative: they suggest node embeddings preserve key topological features of the connectome that are relevant for phenotypic prediction. This comparability both underscores the potential value of connectome embedding approaches and highlights the enduring utility of traditional connectivity measures in predictive modeling. While the similarity in performance may appear trivial at first glance, it carries important implications—namely, that embeddings can be leveraged in future studies without sacrificing predictive fidelity, while also offering advantages in terms of scalability, dimensionality reduction, and potential interpretability.

Our results indicate that node embeddings capture the core predictive signal. In this sense embeddings provide a parsimonious representation of connectivity — not because the models used fewer total predictors (our feature selection procedure always yielded the top 1,000 univariate features in the discovery sample), but because they achieved comparable accuracy without the need for additional feature sets. The lack of performance gains when embeddings were combined with observed or model-implied connectivity suggests that embeddings already capture the core predictive signal. It is in this way that we mean node embeddings represent a parsimonious feature space — they hold their own without the need for additional metrics. Of course, if one *were* interested in building a predictive model with > 1,000 features, then node embeddings provide a more parsimonious framework, in a more traditional sense of the term, for modeling because they reduce the overall number of features (10,560 embedding dimensions overall compared to 61,776 unique connectivity matrix edges).

Added parsimony of either kind not only promises more computationally and statistically efficient model training, but could also promote conceptual interpretability. Embeddings flexibly capture information about individual brain regions and their implicit ties with other regions, meaning that their inclusion in predictive modeling provides utility for compactly uncovering information about both individual parcels and their network connections. Using node embedding gives an analyst the flexibility to focus their interpretation on individual parcels or networks, or compute embedding-implied connectivity and examine the networks that share the highest implied connectivity with the parcels or network identified in the course of predictive modeling.

In our view, this offers unique opportunities for integrative and longitudinal analyses. For example, embeddings derived from functional connectivity models can be compared to those obtained from structural connectivity (Levakov et al., 2021), enabling direct comparisons across imaging modalities (e.g., identifying parcels from a predictive model trained on functional data, then comparing the functional node embedding for the parcels to an embedding from a structural network). Embeddings could also be considered with multivoxel patterns extracted from the brain regions they represent (Guassi Moreira & Silvers, 2025), providing a richer characterization of neural activity. Finally, embeddings lend themselves to longitudinal investigations, allowing researchers to track how nodes evolve within the embedding space over time or across developmental stages. Such analyses could offer a conceptually tractable framework for understanding how brain networks adapt with age, experience, and behavioral outcomes. Together, these advantages position node embeddings as a powerful tool for advancing connectome-based modeling, both in terms of substance use prediction and broader developmental neuroscience questions.

### 4.5 Limitations.

This study has several limitations that represent noteworthy avenues for future investigation. One limitation of this study lies in our cross-validation scheme. While the use of a single train-test split is efficient and ensures demographic matching across splits, it does carry the risk that the specific split chosen could influence the results, potentially yielding numbers that are not fully representative. However, random splits often result in demographic imbalances that could skew predictive accuracy whereas our demographic matching likely mitigates some of this variability. While a solution could involve generating many demographically matched random splits, this process would be computationally intensive and logistically challenging.

Another limitation relates to the fact that the ABCD dataset is not exhaustibly representative of youth around the world and across time. Future studies could leverage other larger datasets, such as the Philadelphia Neurodevelopmental Cohort or ENGIMA studies (Satterthwaite et al., 2014; Thompson et al., 2020). Relatedly, because substance use behavior evolves as policies, economic circumstances, and other similar factors change, it is important to note that our results may not best describe other cohorts.

A further limitation concerns the construction of the substance-use composites across waves. Several variables that were available at Baseline were not collected at the 2-Year Follow-Up, meaning that the composites are not perfectly matched across time. This issue is hardly unique to our study: developmental cognitive neuroscience more broadly has grappled with the tension between ensuring continuity of measurement and adapting measures for developmental appropriateness (Telzer et al., 2018). On one hand, strict continuity offers the cleanest basis for longitudinal comparison but risks asking questions or administering tasks that may not be age-appropriate or informative as youth mature. On the other hand, titrating measures to developmental stage can enhance sensitivity to the most relevant processes at a given age but comes at the cost of strict cross-wave comparability. The field has yet to converge on best practices given the lack of an obviously “correct” solution to this dilemma. In the present study, we prioritized developmental appropriateness within the constraints of the ABCD dataset. As a result, our findings involving the intent and family-developmental history facets should be interpreted with the understanding that differences in predictive performance across waves may partly reflect differences in measurement as much as changes in underlying neurobehavioral processes.

One last consideration involves our approach to controlling for covariates. Some prior approaches residualize outcomes with respect to their covariates before model training, which to our knowledge could inflate predictive accuracy. Residualization removes variance in the outcome that is shared with covariates but not with the predictors, thereby potentially creating an “easier” prediction problem than would be faced in practice. Relatedly, residualization changes the interpretation of the prediction target: instead of modeling a clinically or behaviorally meaningful phenotype, models are trained to predict a statistically adjusted residual that may not map cleanly onto real-world substance-use behaviors. This shift in the interpretation also affects conclusions about magnitude interpretations: Predicting, say, 8% of a residualized outcome means that less variance is necessarily accounted for when predicting a non-residualized outcome. For these reasons, we included covariates directly in the model with our selected features.

## Supplementary Material

Supplement 1

## Figures and Tables

**Table 1.** Study Demographics Split by ABCC Arm.

Variable	Arm 1	Arm 2

*N*	2,929	3,026
Sex (percent female)	52.75%	50.63%

Ethnicity & Race		

Hispanic/Latinx	18.35%	18.46%
Alaska Native	0.00%	0.00%
Asian Indian	0.44%	0.30%
Black	12.51%	13.14%
Chinese	0.55%	0.36%
Filipino	0.31%	0.23%
Guamanian	0.00%	0.00%
Japanese	0.03%	0.03%
Korean	0.14%	0.20%
Native American	0.51%	0.36%
Native Hawaiian	0.00%	0.00%
Other Asian	0.21%	0.30%
Other Pacific Islander	0.10%	0.10%
Samoan	0.10%	0.00%
Vietnamese	0.14%	0.10%
White	69.57%	68.37%
Other Race	2.70%	3.28%
Multi-Racial	11.86%	12.08%
Don’t Know	0.44%	0.63%
Refused	0.27%	0.46%

Socioeconomic Status		

Income	7.50 (2.44)	7.46 (2.23)
Parental Education	17.39 (2.38)	17.34 (2.47)

*Note*. ‘Arm’ refers to the matched group variable from the ABCC collection. Income refers to total combined family income in the prior 12 months. Response options ranged from 1 to 10, corresponding to 1-‘Less than $5,000’, 2-‘ $5,000 - $11,999’, 3-‘$12,000 - $15,999’, 4-‘$16,000 - $24,999’, 5-‘$25,000 - $34,999’, 6-‘$35,000 - $49,999’, 7- ‘$50,000 - $74,999’, 8-‘$75,000 - $99,999’, 9-‘$100,000 - $199,999’, 10-‘$200,000 and greater’. Respondents answering “don’t know” or “decline to answer” were not included in the table mean. Parental education ranged from 0 (‘Never attended/Kindergarten only’) to 21 (‘doctoral degree’); 17 corresponds to ‘Associate degree: Academic program’ and 18 corresponds to ‘Bachelor’s degree (i.e., B.A.)’

**Table 2.** Model accuracies predicting substance use composites at Baseline

***Intent***		

	*Arm 1*	*Arm 2*
PLSR	0.084 (0.70%)	0.104 (1.08%)
RIDGE	0.070 (0.49%)	0.102 (1.04%)
XGB	0.054 (0.29%)	0.078 (0.61%)

***Access***		

	*Arm 1*	*Arm 2*
PLSR	0.064 (0.41%)	0.044 (0.19%)
RIDGE	0.058 (0.34%)	0.069 (0.48%)
XGB	0.043 (0.19%)	0.040 (0.16%)

***Family-Developmental History***		

	*Arm 1*	*Arm 2*
PLSR	0.048 (0.23%)	0.077 (0.60%)
RIDGE	0.081 (0.66%)	0.082 (0.67%)
XGB	0.100 (1.00%)	0.106 (1.12%)

***Impulsivity (UPPS)***		

	*Arm 1*	*Arm 2*
PLSR	0.034 (0.11%)	0.039 (0.15%)
RIDGE	0.040 (0.16%)	0.049 (0.24%)
XGB	0.029 (0.08%)	0.019 (0.04%)

*Note*. ‘Arm’ refers to the arm used for confirmation. ‘XGB’, ‘RIDGE’, and ‘PLSR’ refer to models trained and tested with XGBoost, ridge regression, and partial least squares regression respectively. Entries reflect Pearson correlation’s between fitted and observed values during model testing; parentheticals reflect the square of such values on a percent scale, indicating the proportion of variance explained. Results with the node embedding feature set are copied from the main text here to ease comparison.

**Table 3.** Model accuracies predicting substance use composites at the 2 Year Follow-Up

***Intent***		

	*Arm 1*	*Arm 2*
PLSR	0.099 (0.97%)	0.103 (1.07%)
RIDGE	0.095 (0.91%)	0.115 (1.33%)
XGB	0.184 (3.39%)	0.194 (3.76%)

***Access***		

	*Arm 1*	*Arm 2*
PLSR	0.244 (5.96%)	0.160 (2.56%)
RIDGE	0.154 (2.04%)	0.143 (2.04%)
XGB	0.391 (15.29%)	0.377 (14.21%)

***Family-Developmental History***		

	*Arm 1*	*Arm 2*
PLSR	0.311 (9.65%)	0.379 (14.34%)
RIDGE	0.446 (19.89%)	0.516 (26.58%)
XGB	0.504 (25.40%)	0.543 (29.49%)

***Impulsivity (UPPS)***		

	*Arm 1*	*Arm 2*
PLSR	0.051 (0.26%)	0.063 (0.79%)
RIDGE	0.029 (0.08%)	0.031 (0.10%)
XGB	−0.00 (0.00%)	0.065 (0.79%)

*Note*. ‘Arm’ refers to the arm used for confirmation. ‘XGB’, ‘RIDGE’, and ‘PLSR’ refer to models trained and tested with XGBoost, ridge regression, and partial least squares regression respectively. Entries reflect Pearson correlation’s between fitted and observed values during model testing; parentheticals reflect the square of such values on a percent scale, indicating the proportion of variance explained. Results with the node embedding feature set are copied from the main text here to ease comparison.

## References

[R1] Adkinson B. D., Rosenblatt M., Dadashkarimi J., Tejavibulya L., Jiang R., Noble S., & Scheinost D. (2024). Brain-phenotype predictions of language and executive function can survive across diverse real-world data: Dataset shifts in developmental populations. Developmental Cognitive Neuroscience, 70, 101464. 10.1016/j.dcn.2024.101464 39447452 PMC11538622

[R2] Canario E., Chen D., & Biswal B. (2021). A review of resting-state fMRI and its use to examine psychiatric disorders. Psychoradiology, 1(1), 42–53. 10.1093/psyrad/kkab003 38665309 PMC10917160

[R3] Caouette J. D., & Feldstein Ewing S. W. (2017). Four mechanistic models of peer influence on adolescent cannabis use. Current Addiction Reports, 4(2), 90–99. 10.1007/s40429-017-0144-0 29104847 PMC5663303

[R4] Casey B. J., Cannonier T., Conley M. I., Cohen A. O., Barch D. M., Heitzeg M. M., Soules M. E., Teslovich T., Dellarco D. V., Garavan H., Orr C. A., Wager T. D., Banich M. T., Speer N. K., Sutherland M. T., Riedel M. C., Dick A. S., Bjork J. M., Thomas K. M., … ABCD Imaging Acquisition Workgroup. (2018). The Adolescent Brain Cognitive Development (ABCD) study: Imaging acquisition across 21 sites. Developmental Cognitive Neuroscience, 32, 43–54. 10.1016/j.dcn.2018.03.001 29567376 PMC5999559

[R5] Centers for Disease Control and Prevention. (2020). YOUTH RISK BEHAVIOR SURVEY DATA SUMMARY & TRENDS REPORT 2007–2017.

[R6] Cervantes E. A., Miller W. R., & Tonigan J. S. (1994). Comparison of Timeline Follow-Back and Averaging Methods for Quantifying Alcohol Consumption in Treatment Research. Assessment, 1(1), 23–30. 10.1177/1073191194001001004 9463496

[R7] Cunningham R. M., Walton M. A., & Carter P. M. (2018). The major causes of death in children and adolescents in the united states. The New England Journal of Medicine, 379(25), 2468–2475. 10.1056/NEJMsr1804754 30575483 PMC6637963

[R8] Dick D. M., Pagan J. L., Viken R., Purcell S., Kaprio J., Pulkkinen L., & Rose R. J. (2007). Changing environmental influences on substance use across development. Twin Research and Human Genetics, 10(2), 315–326. 10.1375/twin.10.2.315 17564520 PMC1905859

[R9] Do K. T., Guassi Moreira J. F., & Telzer E. H. (2017). But is helping you worth the risk? Defining Prosocial Risk Taking in adolescence. Developmental Cognitive Neuroscience, 25, 260–271. 10.1016/j.dcn.2016.11.008 28063823 PMC5461219

[R10] Duell N., Steinberg L., Icenogle G., Chein J., Chaudhary N., Di Giunta L., Dodge K. A., Fanti K. A., Lansford J. E., Oburu P., Pastorelli C., Skinner A. T., Sorbring E., Tapanya S., Uribe Tirado L. M., Alampay L. P., Al-Hassan S. M., Takash H. M. S., Bacchini D., & Chang L. (2018). Age patterns in risk taking across the world. Journal of Youth and Adolescence, 47(5), 1052–1072. 10.1007/s10964-017-0752-y 29047004 PMC5878702

[R11] Duell N., & Steinberg L. (2019). Positive risk taking in adolescence. Child Development Perspectives, 13(1), 48–52. 10.1111/cdep.12310 30774707 PMC6371981

[R12] Feczko E., Conan G., Marek S., Tervo-Clemens B., Cordova M., Doyle O., Earl E., Perrone A., Sturgeon D., Klein R., Harman G., Kilamovich D., Hermosillo R., Miranda-Dominguez O., Adebimpe A., Bertolero M., Cieslak M., Covitz S., Hendrickson T., … Fair D. A. (2021). Adolescent brain cognitive development (ABCD) community MRI collection and utilities. BioRxiv. 10.1101/2021.07.09.451638

[R13] Fujimoto K., & Valente T. W. (2012). Social network influences on adolescent substance use: disentangling structural equivalence from cohesion. Social Science & Medicine, 74(12), 1952–1960. 10.1016/j.socscimed.2012.02.009 22475405 PMC3354645

[R14] Gao S., Greene A. S., Constable R. T., & Scheinost D. (2019). Combining multiple connectomes improves predictive modeling of phenotypic measures. Neuroimage, 201, 116038. 10.1016/j.neuroimage.2019.116038 31336188 PMC6765422

[R15] Garavan H., Bartsch H., Conway K., Decastro A., Goldstein R. Z., Heeringa S., Jernigan T., Potter A., Thompson W., & Zahs D. (2018). Recruiting the ABCD sample: Design considerations and procedures. Developmental Cognitive Neuroscience, 32, 16–22. 10.1016/j.dcn.2018.04.004 29703560 PMC6314286

[R16] Garofoli M. (2020). Adolescent Substance Abuse. Primary Care, 47(2), 383–394. 10.1016/j.pop.2020.02.013 32423721

[R17] Goldberg Y., & Levy O. (2014). word2vec Explained: deriving Mikolov et al.’s negative-sampling word-embedding method. ArXiv. 10.48550/arxiv.1402.3722

[R18] Gordon E. M., Laumann T. O., Adeyemo B., Huckins J. F., Kelley W. M., & Petersen S. E. (2016). Generation and Evaluation of a Cortical Area Parcellation from Resting-State Correlations. Cerebral Cortex, 26(1), 288–303. 10.1093/cercor/bhu239 25316338 PMC4677978

[R19] Gratton C., Dworetsky A., Adeyemo B., Seitzman B. A., Smith D. M., Petersen S. E., & Neta M. (2022). The cingulo-opercular network is composed of two distinct sub-systems. BioRxiv. 10.1101/2022.09.16.508254

[R20] Guassi Moreira J. F., & Silvers J. A. (2025). Multi-Voxel Pattern Analysis for Developmental Cognitive Neuroscientists. Developmental Cognitive Neuroscience. 10.1016/j.dcn.2025.101555 PMC12002837 40188575

[R21] Hussong A. M. (2002). Differentiating peer contexts and risk for adolescent substance use. Journal of Youth and Adolescence, 31(3), 207–220. 10.1023/A:1015085203097

[R22] Hyon R., Chavez R. S., Chwe J. A. H., Wheatley T., Kleinbaum A. M., & Parkinson C. (2022). White matter connectivity in brain networks supporting social and affective processing predicts real-world social network characteristics. Communications Biology, 5(1), 1048. 10.1038/s42003-022-03655-8 36192629 PMC9529948

[R23] Hyon R., Youm Y., Kim J., Chey J., Kwak S., & Parkinson C. (2020). Similarity in functional brain connectivity at rest predicts interpersonal closeness in the social network of an entire village. Proceedings of the National Academy of Sciences of the United States of America, 117(52), 33149–33160. 10.1073/pnas.2013606117 33318188 PMC7777022

[R24] Johnston L., Miech R., O’Malley P., Bachman J., Schulenberg J., & Patrick M. (2019). Monitoring the Future national survey results on drug use, 1975–2018: Overview, key findings on adolescent drug use. University of Michigan Institute for Social Research. 10.3998/2027.42/150621

[R25] Levakov G., Faskowitz J., Avidan G., & Sporns O. (2021). Mapping individual differences across brain network structure to function and behavior with connectome embedding. Neuroimage, 242, 118469. 10.1016/j.neuroimage.2021.118469 34390875 PMC8464439

[R26] Lilja J., Larsson S., Wilhelmsen B. U., & Hamilton D. (2003). Perspectives on preventing adolescent substance use and misuse. Substance Use & Misuse, 38(10), 1491–1530. 10.1081/ja-120023395 14509548

[R27] Lisdahl K. M., & Price J. S. (2012). Increased marijuana use and gender predict poorer cognitive functioning in adolescents and emerging adults. Journal of the International Neuropsychological Society, 18(4), 678–688. 10.1017/S1355617712000276 22613255 PMC3956124

[R28] Mallard T. T., Doorley J., Esposito-Smythers C. L., & McGeary J. E. (2016). Dopamine D4 receptor VNTR polymorphism associated with greater risk for substance abuse among adolescents with disruptive behavior disorders: Preliminary results. The American Journal on Addictions, 25(1), 56–61. 10.1111/ajad.12320 26688118

[R29] Marek S., Tervo-Clemmens B., Calabro F. J., Montez D. F., Kay B. P., Hatoum A. S., Donohue M. R., Foran W., Miller R. L., Hendrickson T. J., Malone S. M., Kandala S., Feczko E., Miranda-Dominguez O., Graham A. M., Earl E. A., Perrone A. J., Cordova M., Doyle O., … Dosenbach N. U. F. (2022). Reproducible brain-wide association studies require thousands of individuals. Nature, 603(7902), 654–660. 10.1038/s41586-022-04492-9 35296861 PMC8991999

[R30] Marek S., Tervo-Clemmens B., Nielsen A. N., Wheelock M. D., Miller R. L., Laumann T. O., Earl E., Foran W. W., Cordova M., Doyle O., Perrone A., Miranda-Dominguez O., Feczko E., Sturgeon D., Graham A., Hermosillo R., Snider K., Galassi A., Nagel B. J., … Dosenbach N. U. F. (2019). Identifying reproducible individual differences in childhood functional brain networks: An ABCD study. Developmental Cognitive Neuroscience, 40, 100706. 10.1016/j.dcn.2019.100706 31614255 PMC6927479

[R31] McGue M., Elkins I., & Iacono W. G. (2000). Genetic and environmental influences on adolescent substance use and abuse. American Journal of Medical Genetics. 10.1002/1096-8628(20001009)96:5<671::aid-ajmg14>3.0.co;2-w 11054776

[R32] Mikolov T., Chen K., Corrado G., & Dean J. (2013). Efficient estimation of word representations in vector space. ArXiv. 10.48550/arxiv.1301.3781

[R33] Ooi L. Q. R., Orban C., Zhang S., Nichols T. E., Tan T. W. K., Kong R., Marek S., Dosenbach N. U. F., Laumann T. O., Gordon E. M., Yap K. H., Ji F., Chong J. S. X., Chen C., An L., Franzmeier N., Roemer-Cassiano S. N., Hu Q., Ren J., … Alzheimer’s Disease Neuroimaging Initiative. (2025). Longer scans boost prediction and cut costs in brain-wide association studies. Nature. 10.1038/s41586-025-09250-1 PMC12367542 40670782

[R34] Petersen S. E., Seitzman B. A., Nelson S. M., Wig G. S., & Gordon E. M. (2024). Principles of cortical areas and their implications for neuroimaging. Neuron. 10.1016/j.neuron.2024.05.008 PMC13339775 38834069

[R35] Peverill M., Dirks M. A., Narvaja T., Herts K. L., Comer J. S., & McLaughlin K. A. (2021). Socioeconomic status and child psychopathology in the United States: A meta-analysis of population-based studies. Clinical Psychology Review, 83, 101933. 10.1016/j.cpr.2020.101933 33278703 PMC7855901

[R36] Rogers C. J., Pakdaman S., Forster M., Sussman S., Grigsby T. J., Victoria J., & Unger J. B. (2022). Effects of multiple adverse childhood experiences on substance use in young adults: A review of the literature. Drug and Alcohol Dependence, 234, 109407. 10.1016/j.drugalcdep.2022.109407 35306395

[R37] Ruchkin V., Koposov R., Oreland L., af.Klinteberg B., & Grigorenko E. L. (2021). Dopamine-related receptors, substance dependence, behavioral problems and personality among juvenile delinquents. Personality and Individual Differences, 169, 109849. 10.1016/j.paid.2020.109849

[R38] Rudolph M. D., Graham A. M., Feczko E., Miranda-Dominguez O., Rasmussen J. M., Nardos R., Entringer S., Wadhwa P. D., Buss C., & Fair D. A. (2018). Maternal IL-6 during pregnancy can be estimated from newborn brain connectivity and predicts future working memory in offspring. Nature Neuroscience, 21(5), 765–772. 10.1038/s41593-018-0128-y 29632361 PMC5920734

[R39] Satterthwaite T. D., Elliott M. A., Ruparel K., Loughead J., Prabhakaran K., Calkins M. E., Hopson R., Jackson C., Keefe J., Riley M., Mentch F. D., Sleiman P., Verma R., Davatzikos C., Hakonarson H., Gur R. C., & Gur R. E. (2014). Neuroimaging of the Philadelphia neurodevelopmental cohort. Neuroimage, 86, 544–553. 10.1016/j.neuroimage.2013.07.064 23921101 PMC3947233

[R40] Schettino M., Mauti M., Parrillo C., Ceccarelli I., Giove F., Napolitano A., Ottaviani C., Martelli M., & Orsini C. (2024). Resting-state brain activation patterns and network topology distinguish human sign and goal trackers. Translational Psychiatry, 14(1), 446. 10.1038/s41398-024-03162-w 39438457 PMC11496639

[R41] Seitzman B. A., Snyder A. Z., Leuthardt E. C., & Shimony J. S. (2019). The state of resting state networks. Topics in Magnetic Resonance Imaging: TMRI, 28(4), 189–196. 10.1097/RMR.0000000000000214 31385898 PMC6686880

[R42] Shen X., Finn E. S., Scheinost D., Rosenberg M. D., Chun M. M., Papademetris X., & Constable R. T. (2017). Using connectome-based predictive modeling to predict individual behavior from brain connectivity. Nature Protocols, 12(3), 506–518. 10.1038/nprot.2016.178 28182017 PMC5526681

[R43] Silberg J., Rutter M., D’Onofrio B., & Eaves L. (2003). Genetic and environmental risk factors in adolescent substance use. Journal of Child Psychology and Psychiatry, and Allied Disciplines, 44(5), 664–676. 10.1111/1469-7610.00153 12831111

[R44] Skowronek M. H., Laucht M., Hohm E., Becker K., & Schmidt M. H. (2006). Interaction between the dopamine D4 receptor and the serotonin transporter promoter polymorphisms in alcohol and tobacco use among 15-year-olds. Neurogenetics, 7(4), 239–246. 10.1007/s10048-006-0050-4 16819620

[R45] Spisak T., Bingel U., & Wager T. D. (2023). Multivariate BWAS can be replicable with moderate sample sizes. Nature, 615(7951), E4–E7. 10.1038/s41586-023-05745-x 36890392 PMC9995263

[R46] Steen J. A. (2010). A multilevel study of the role of environment in adolescent substance use. Journal of Child & Adolescent Substance Abuse, 19(5), 359–371. 10.1080/1067828X.2010.502479

[R47] Tervo-Clemmens B., Karim Z. A., Khan S. Z., Ravindranath O., Somerville L. H., Schuster R. M., Gilman J. M., & Evins A. E. (2024). The Developmental Timing but Not Magnitude of Adolescent Risk-Taking Propensity Is Consistent Across Social, Environmental, and Psychological Factors. The Journal of Adolescent Health, 74(3), 613–616. 10.1016/j.jadohealth.2023.11.001 38085210 PMC12372624

[R48] Thompson P. M., Jahanshad N., Ching C. R. K., Salminen L. E., Thomopoulos S. I., Bright J., Baune B. T., Bertolín S., Bralten J., Bruin W. B., Bülow R., Chen J., Chye Y., Dannlowski U., de Kovel C. G. F., Donohoe G., Eyler L. T., Faraone S. V., Favre P., … ENIGMA Consortium. (2020). ENIGMA and global neuroscience: A decade of large-scale studies of the brain in health and disease across more than 40 countries. Translational Psychiatry, 10(1), 100. 10.1038/s41398-020-0705-1 32198361 PMC7083923

[R49] Tomczyk S., Isensee B., & Hanewinkel R. (2016). Latent classes of polysubstance use among adolescents-a systematic review. Drug and Alcohol Dependence, 160, 12–29. 10.1016/j.drugalcdep.2015.11.035 26794683

[R50] Toumbourou J. W., Stockwell T., Neighbors C., Marlatt G. A., Sturge J., & Rehm J. (2007). Interventions to reduce harm associated with adolescent substance use. The Lancet, 369(9570), 1391–1401. 10.1016/S0140-6736(07)60369-9 17448826

[R51] Uddin L. Q., Castellanos F. X., & Menon V. (2024). Resting state functional brain connectivity in child and adolescent psychiatry: where are we now? Neuropsychopharmacology, 50(1), 196–200. 10.1038/s41386-024-01888-1 38778158 PMC11525794

[R52] Uddin L. Q., Yeo B. T. T., & Spreng R. N. (2019). Towards a Universal Taxonomy of Macro-scale Functional Human Brain Networks. Brain Topography, 32(6), 926–942. 10.1007/s10548-019-00744-6 31707621 PMC7325607

[R53] Vergunst F., Chadi N., Orri M., Brousseau-Paradis C., Castellanos-Ryan N., Séguin J. R., Vitaro F., Nagin D., Tremblay R. E., & Côté S. M. (2022). Trajectories of adolescent poly-substance use and their long-term social and economic outcomes for males from low-income backgrounds. European Child & Adolescent Psychiatry, 31(11), 1729–1738. 10.1007/s00787-021-01810-w 34059981

[R54] Wu J., Li J., Eickhoff S. B., Scheinost D., & Genon S. (2023). The challenges and prospects of brain-based prediction of behaviour. Nature Human Behaviour, 7(8), 1255–1264. 10.1038/s41562-023-01670-1 37524932

[R55] Yarkoni T., & Westfall J. (2017). Choosing prediction over explanation in psychology: lessons from machine learning. Perspectives on Psychological Science, 12(6), 1100–1122. 10.1177/1745691617693393 28841086 PMC6603289

