# Supplementary material for: Connectome-Based Predictive Modeling of Concurrent and Prospective Substance Use in Adolescence
Source: bioRxiv. 2025 Sep 4:2025.09.01.673428. Preprint. [Version 1] doi: 10.1101/2025.09.01.673428 (PMC12424972; doi:10.1101/2025.09.01.673428)
Supplement: Supplement 1 [file NIHPP2025.09.01.673428v1-supplement-1.pdf]

bioRxiv preprint doi: https://doi.org/10.1101/2025.09.01.673428; this version posted September 4, 2025. The copyright holder for this preprint (which was not certified by peer review) is the author/funder, who has granted bioRxiv a license to display the preprint in perpetuity. It is made available under aCC-BY 4.0 International license.

Supplement

S1. Connectome Embedding Model Details.

The node2vec algorithm is based on the seminal word2vec algorithm (Mikolov et al., 2013), originally developed to help estimate vectorized representations of naturalistic speech. While this model was initially used to predict words in a sequence of text from their context, or predicting the context from a target word (Goldberg & Levy, 2014), there is no intrinsic requirement that constrains the algorithm to naturalistic speech and it can be applied to estimate vector embeddings for nodes in a graph object.

The algorithm learns two weight matrices – one that defines the embedding space and contains the vector representations for each node (*W*), and a second one that transforms the embedding layer to the output layer (*W'*). Stochastic gradient descent is used to update these two matrices by minimizing the negative log loss function. Here we used a sliding window of s = 3 nodes, meaning that the context nodes were comprised of the s nodes that appeared before the target node *and* the s nodes that appeared after it. We simulated o = 800 random walk sequences, each of length l = 20. We specified 30 latent dimensions such that an embedding for a given node contained 30 values.

The random sequences were parameterized such that we applied constants to control the behavior of the walks in order to balance local- vs global-bias. Briefly, communication within functional brain connectivity networks is thought to be driven by local connections between nodes (in a geodesic manner, not necessarily a spatial one). Importantly, the node2vec algorithm assumes information about a node's local neighborhood, which means the simulated random walks needed to reflect this in order to promote biological and computational compatibility. Consequently, two parameters in the random walk simulation were set to ensure locally-biased sequences. A p parameter sets the return probability (walker immediately returns to the node in the next step) and a q parameter sets the in-out probability (walker visit a non-connected node in the next step). These constants are divided by the sum of edges depending on the node for the current step in the walk (p if the next candidate node is the same as the previous node; q if the next candidate node is fully disconnected). Thus, we set p = 0.1 and q = 1.6 to promote locally-biased networks. These parameters are consistent with prior work using these methods (Rosenthal et al., 2018).

This model fitting procedure was fit to each subject's functional connectivity data. A random subset of 50 participants had their functional connectivity matrices averaged and used to fit a single connectome embedding model. The embedding space for this particular model was used as a reference space by which all subject-level embedding spaces were subsequently aligned (Levakov et al., 2021). Whereas the embedding similarity of a given node pair is comparable between embedding spaces, the absolute value of the embeddings is not. To ensure comparability between spaces, individual subject embeddings must be aligned to a reference embedding space. This is achieved by multiplying *W'* of the target reference space with the embedding space for the desired participant (*W*).

Next, as a sanity check, we wanted to ensure the embedding model fit properly. We did this by estimating the relationship between observed connectivity and model-

bioRxiv preprint doi: https://doi.org/10.1101/2025.09.01.673428; this version posted September 4, 2025. The copyright holder for this preprint (which was not certified by peer review) is the author/funder, who has granted bioRxiv a license to display the preprint in perpetuity. It is made available under aCC-BY 4.0 International license.

implied connectivity that is derived by taking the similarity between two parcel's embedding vectors. Practically, this means that we took the correlation between each participant's observed connectivity (correlation matrix comprised of all possible pairwise correlations among the parcel dense time series) and the model implied connectivity obtained by taking the cosine similarity among vector embeddings. This step helped gauge the degree to which the connectome embedding model performs as an accurate representation of the connectivity. Results are shown in Supplementary Figure 1, indicating that the connectome embedding models accurately capture overall rs-fMRI connectivity across participants.

S2. Substance Use Composite Variable Details.

The intent composite variable was created in the following manner. First, using data from the baseline timeline follow-back assessment, we created a summed composite of the items indicating whether youths had *heard* about a particular substance (tlfb_ + alc, tob, mj, mj_synth, bitta, caff, inhalant, rx_misuse, list_yes_no). We applied the same procedure to the variables asking youths whether they had *tried* a particular substance (tlfb_alc_sip, tlfb_tob_puff, tlfb_*_use, tlfb_*_use_type___*) as well as the variables tapping regular use of each substance (tlfb_*_reg). These three sum score composites (sub_use_heard, sub_use_tried, sub_use_reg) were concatenated with additional variables that tapped alcohol, nicotine, and caffeine use (path_acl_youth[1-8], su_isip_1_calc, su_isip_1b_2_calc, first_nicotine_1, first_nicotine_3, first_mj_1b, su_caff_ss_sum_calc). The final intent composite was created by averaging scores of each individual variable in this concatenated variable set.

The access composite variable was created by computing the mean of the following variables for each participant. peer_deviance_1_4bbe5d, peer_deviance_2_dd1457, peer_deviance_3_e1ec2e, peer_deviance_4_b6c588, peer_deviance_5_bffa44, peer_deviance_6_69562e, peer_deviance_7_beb683, peer_deviance_8_35702e, peer_deviance_9_6dd4ef, parent_rules_q1, parent_rules_q1a, parent_rules_q[2-9], su_risk_p_[1-9].

Finally, the family-developmental history (fdhx) composite was created in the same manner using the following variables. asr_q06_p, asr_q90_p, asr_q124_p, asr_q126_p, scrn_hr_smoke, fam_history_5_yes_no, famhx_4_p, devhx_8_tobacco, devhx_8_alcohol, devhx_8_marijuana, devhx_9_tobacco, devhx_9_alcohol, devhx_9_marijuana.

All items are presented in table form in Supplementary Tables 1A-C; the tables indicate item availability by wave.

bioRxiv preprint doi: https://doi.org/10.1101/2025.09.01.673428; this version posted September 4, 2025. The copyright holder for this preprint (which was not certified by peer review) is the author/funder, who has granted bioRxiv a license to display the preprint in perpetuity. It is made available under aCC-BY 4.0 International license.

Supplementary Figure 1. Distribution of observed and model implied connectivity correlations.

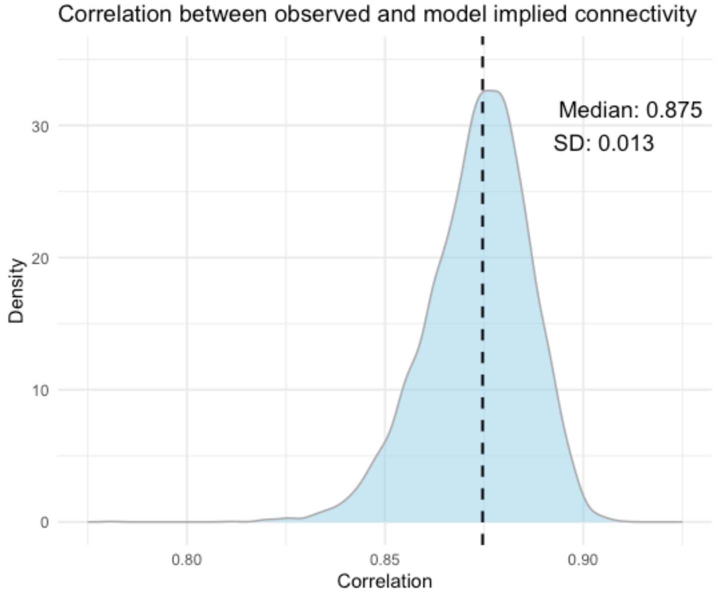

bioRxiv preprint doi: https://doi.org/10.1101/2025.09.01.673428; this version posted September 4, 2025. The copyright holder for this preprint (which was not certified by peer review) is the author/funder, who has granted bioRxiv a license to display the preprint in perpetuity. It is made available under aCC-BY 4.0 International license.

Supplementary Figure 2. Prediction accuracies split by outcome, inclusive of all feature sets

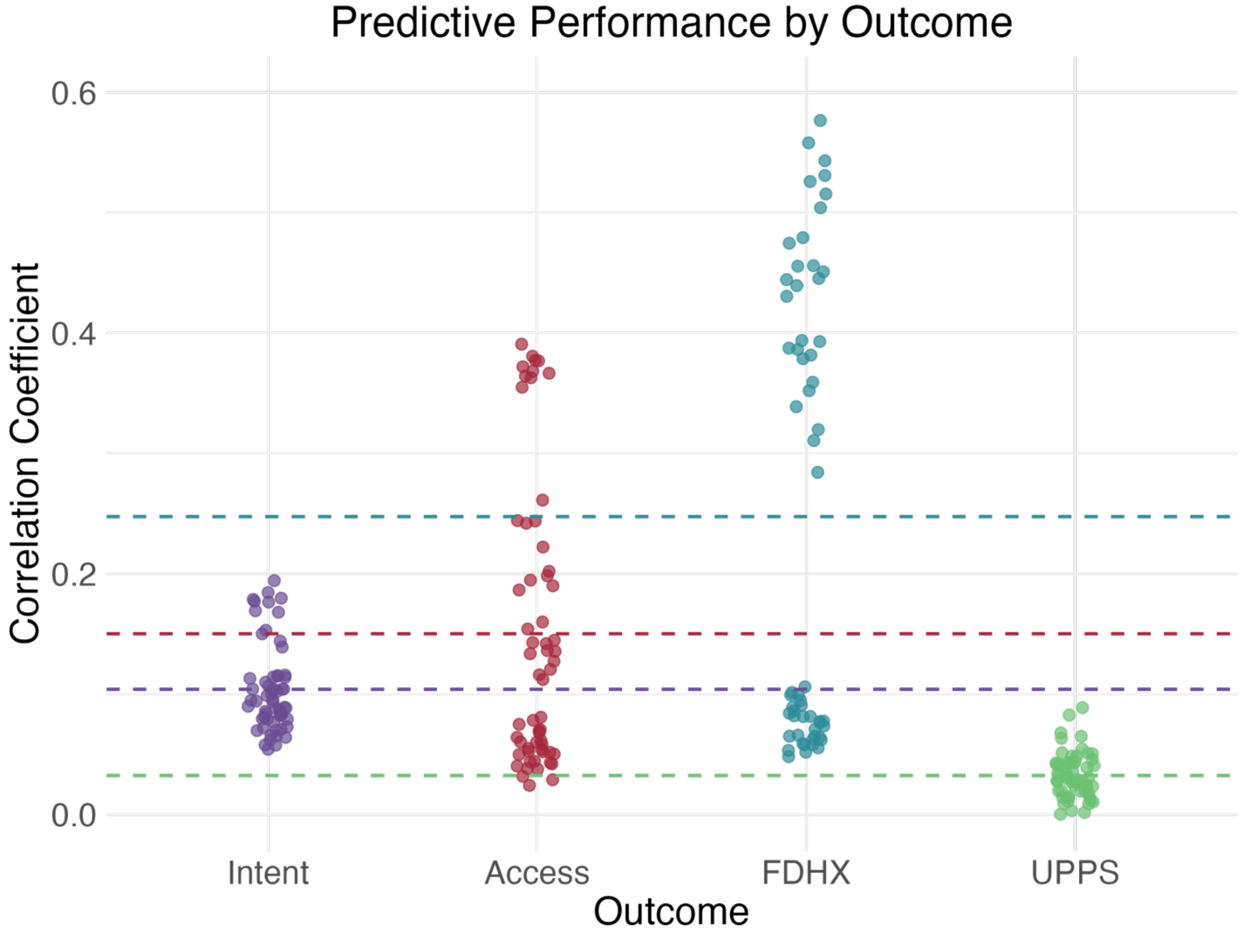

Note. ‘FDHX’ refers to family-developmental history; UPPS refers to impulsivity measure with the UPPS-P Impulsive Behavior Scale. Averages among all correlations in each category is depicted with the hashed lines.

bioRxiv preprint doi: https://doi.org/10.1101/2025.09.01.673428; this version posted September 4, 2025. The copyright holder for this preprint (which was not certified by peer review) is the author/funder, who has granted bioRxiv a license to display the preprint in perpetuity. It is made available under aCC-BY 4.0 International license.

Supplementary Figure 3. Prediction accuracies split by all feature sets

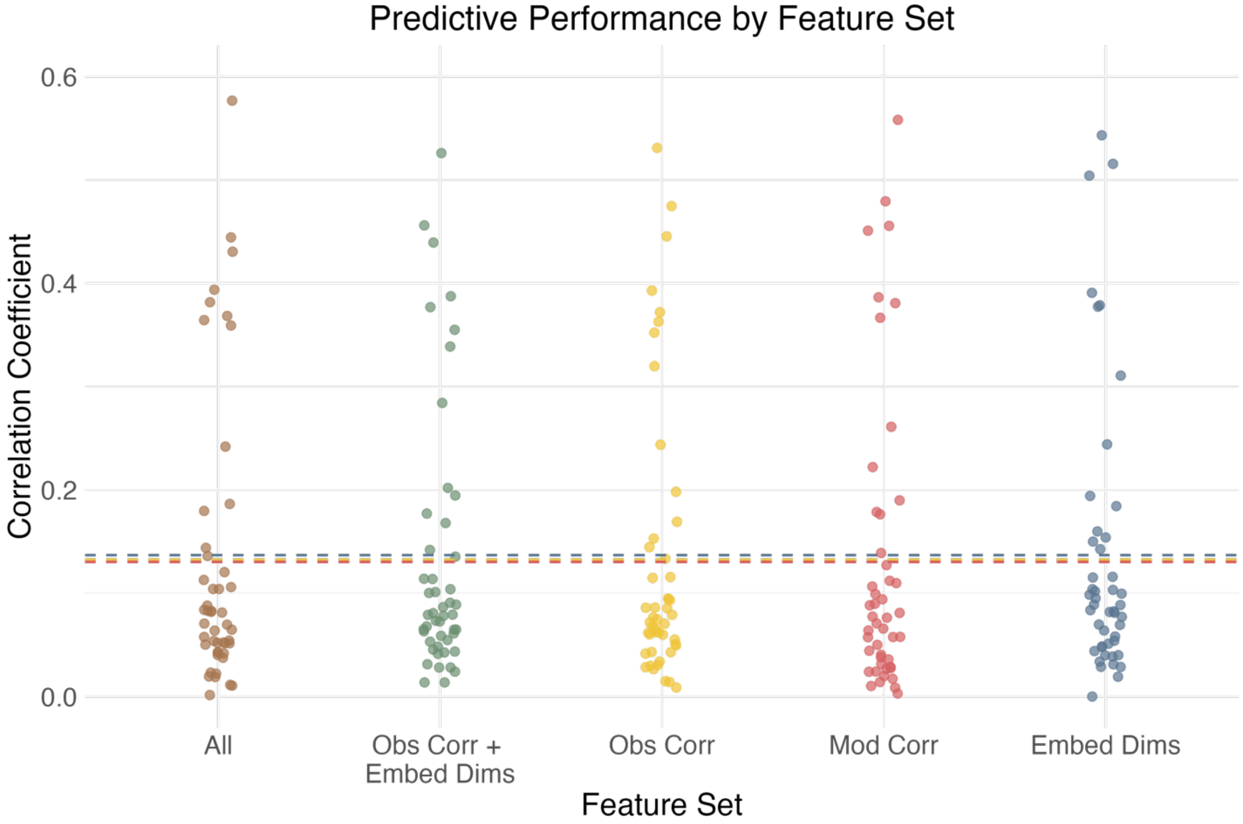

Note. 'Obs Corr', 'Mod Corr' and 'Embed Dims' refer to observed connectivity, model implied connectivity, and node embedding dimensions respectively. 'All' refers to a feature set with all three connectivity metrics. Averages among all correlations in each category is depicted with the hashed lines.

bioRxiv preprint doi: https://doi.org/10.1101/2025.09.01.673428; this version posted September 4, 2025. The copyright holder for this preprint (which was not certified by peer review) is the author/funder, who has granted bioRxiv a license to display the preprint in perpetuity. It is made available under aCC-BY 4.0 International license.

Supplementary Figure 4. Prediction accuracies by model types, inclusive of all feature sets

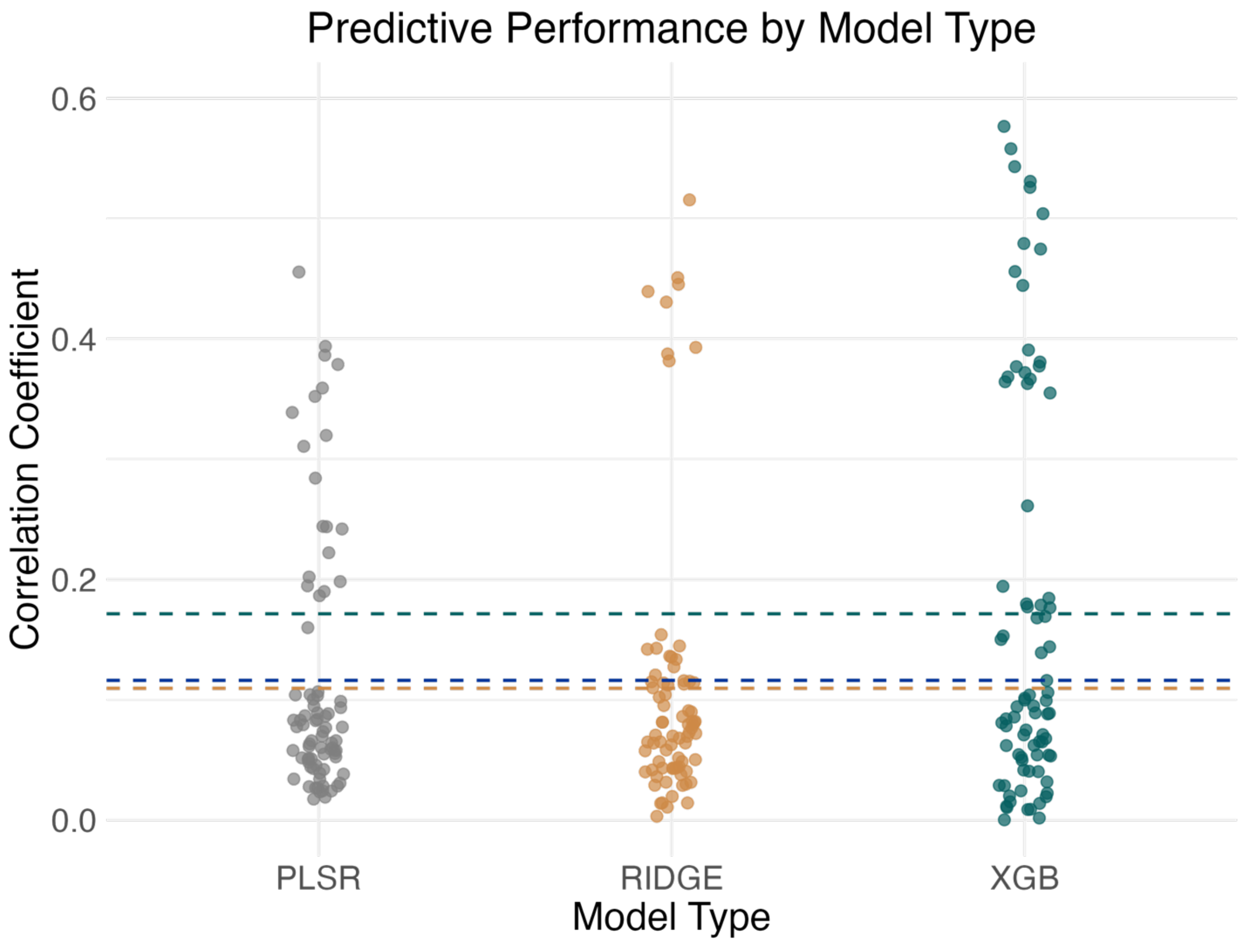

Note. 'XGB', 'RIDGE', and 'PLSR' refer to models trained and tested with XGBoost, ridge regression, and partial least squares regression respectively. Averages among all correlations in each category is depicted with the hashed lines.

bioRxiv preprint doi: https://doi.org/10.1101/2025.09.01.673428; this version posted September 4, 2025. The copyright holder for this preprint (which was not certified by peer review) is the author/funder, who has granted bioRxiv a license to display the preprint in perpetuity. It is made available under aCC-BY 4.0 International license.

Supplementary Figure 5. Prediction accuracies plotted by which arm served as the confirmation dataset (top: inclusive of all feature sets, bottom: model specifications with node embedding feature set only)

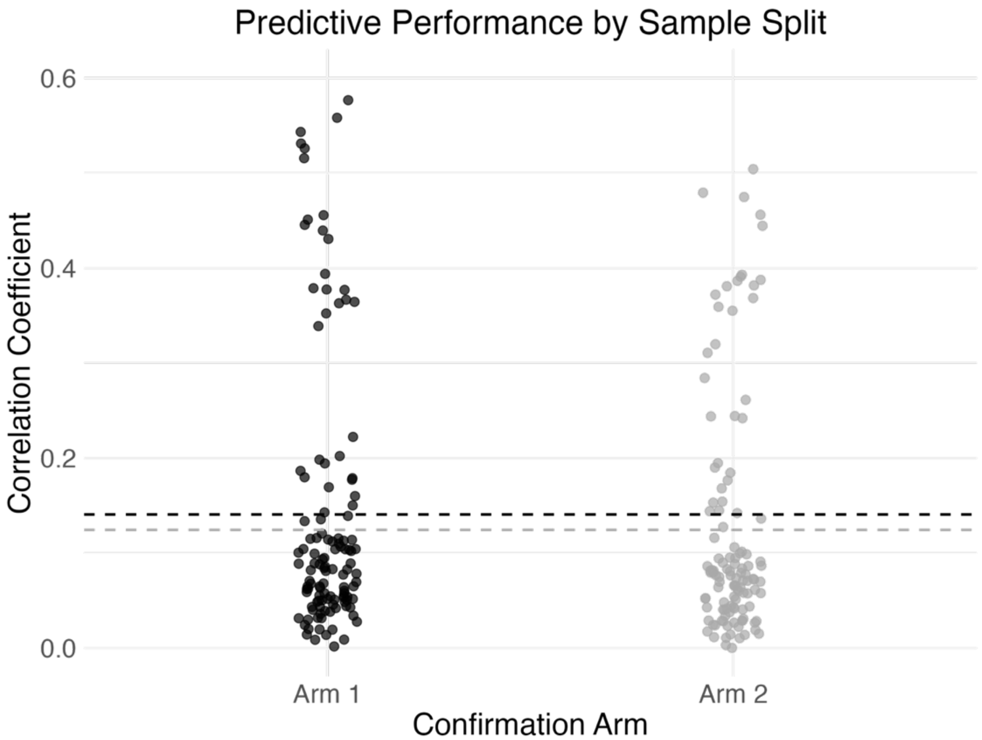

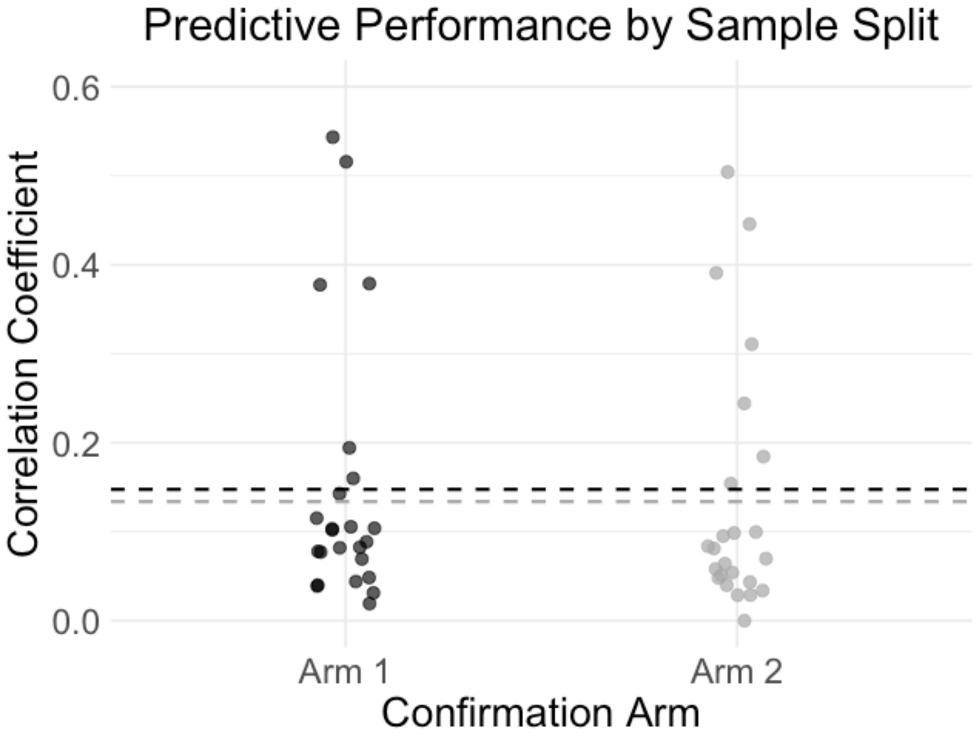

bioRxiv preprint doi: https://doi.org/10.1101/2025.09.01.673428; this version posted September 4, 2025. The copyright holder for this preprint (which was not certified by peer review) is the author/funder, who has granted bioRxiv a license to display the preprint in perpetuity. It is made available under aCC-BY 4.0 International license.

Supplementary Table 1A. Full set of *intent* variables used in the composite

tlfb_alc	tlfb_mj_synth_use	tlfb_hall_use_type___8	tlfb_edible_reg
tlfb_tob	tlfb_coc_use	tlfb_hall_use_type___10	tlfb_mj_conc_reg
tlfb_mj	tlfb_bsalts_use	tlfb_inhalant_use_type___1	tlfb_mj_drink_reg
tlfb_mj_synth	tlfb_meth_use	tlfb_inhalant_use_type___2	tlfb_tincture_reg
tlfb_bitta	tlfb_mdma_use	tlfb_inhalant_use_type___3	tlfb_mj_synth_reg
tlfb_caff	tlfb_ket_use	tlfb_inhalant_use_type___4	tlfb_coc_reg
tlfb_inhalant	tlfb_ghb_use	tlfb_inhalant_use_type___5	tlfb_bsalts_reg
tlfb_rx_misuse	tlfb_opi_use	tlfb_inhalant_use_type___6	tlfb_meth_reg
tlfb_list_yes_no	tlfb_hall_use	tlfb_inhalant_use_type___7	tlfb_mdma_reg
tlfb_alc_sip	tlfb_shrooms_use	tlfb_inhalant_use_type___8	tlfb_ket_reg
tlfb_alc_use	tlfb_salvia_use	su_isip_1_calc	tlfb_ghb_reg
tlfb_tob_puff	tlfb_steroids_use	su_isip_1b_2_calc	tlfb_opi_reg
tlfb_cig_use	tlfb_bitta_use	tlfb_alc_reg	tlfb_hall_reg
tlfb_ecig_use	tlfb_sniff_use	tlfb_alc_calc_reg	tlfb_shrooms_reg
tlfb_chew_use	tlfb_inhalant_use	first_nicotine_1	tlfb_salvia_reg
tlfb_cigar_use	tlfb_amp_use	first_nicotine_3	tlfb_steroids_reg
tlfb_hookah_use	tlfb_tranq_use	tlfb_cig_reg	tlfb_bitta_reg
tlfb_pipes_use	tlfb_vicodin_use	tlfb_ecig_reg	tlfb_inhalant_reg
tlfb_nicotine_use	tlfb_cough_use	tlfb_chew_reg	tlfb_amp_reg
tlfb_mj_puff	tlfb_other_use	tlfb_cigar_reg	tlfb_tranq_reg
tlfb_mj_use	tlfb_hall_use_type___1	tlfb_hookah_reg	tlfb_vicodin_reg
tlfb_blunt_use	tlfb_hall_use_type___2	tlfb_pipes_reg	tlfb_cough_reg
tlfb_edible_use	tlfb_hall_use_type___3	tlfb_nicotine_reg	su_caff_ss_sum_calc
tlfb_mj_conc_use	tlfb_hall_use_type___4	first_mj_1b	path_alc_youth1
tlfb_mj_drink_use	tlfb_hall_use_type___6	tlfb_mj_reg	path_alc_youth2
tlfb_tincture_use	tlfb_hall_use_type___7	tlfb_blunt_reg	path_alc_youth3

bioRxiv preprint doi: https://doi.org/10.1101/2025.09.01.673428; this version posted September 4, 2025. The copyright holder for this preprint (which was not certified by peer review) is the author/funder, who has granted bioRxiv a license to display the preprint in perpetuity. It is made available under aCC-BY 4.0 International license.

Supplementary Table 1A, continued

path_alc_youth4
path_alc_youth5
path_alc_youth6
path_alc_youth7
path_alc_youth8
path_alc_youth9

Note. Cells highlighted in blue were collected at both timepoints, cells with no highlighting were only collected at Baseline. 'tlfb' references the timeline follow-back and life use inventory items. 'path' references the PATH inventory which measures youth's curiosity to use alcohol. The 'su_caff_ss_sum_calc' refers to caffeine usage.

bioRxiv preprint doi: https://doi.org/10.1101/2025.09.01.673428; this version posted September 4, 2025. The copyright holder for this preprint (which was not certified by peer review) is the author/funder, who has granted bioRxiv a license to display the preprint in perpetuity. It is made available under aCC-BY 4.0 International license.

Supplementary Table 1B. Full set of *Access* variables used in the composite

peer_deviance_1_4bbe5d	peer_deviance_8_35702e	parent_rules_q5	su_risk_p_3
peer_deviance_2_dd1457	peer_deviance_9_6dd4ef	parent_rules_q6	su_risk_p_4
peer_deviance_3_e1ec2e	parent_rules_q1	parent_rules_q7	su_risk_p_5
peer_deviance_4_b6c588	parent_rules_q1a	parent_rules_q8	su_risk_p_6
peer_deviance_5_bffa44	parent_rules_q2	parent_rules_q9	su_risk_p_7
peer_deviance_6_69562e	parent_rules_q3	su_risk_p_1	su_risk_p_8
peer_deviance_7_beb683	parent_rules_q4	su_risk_p_2	su_risk_p_9

Note. Cells highlighted in blue were collected at both timepoints, cells with no highlighting were only collected at Baseline.

bioRxiv preprint doi: https://doi.org/10.1101/2025.09.01.673428; this version posted September 4, 2025. The copyright holder for this preprint (which was not certified by peer review) is the author/funder, who has granted bioRxiv a license to display the preprint in perpetuity. It is made available under aCC-BY 4.0 International license.

*Supplementary Table 1C***.** Full set of *FDHX* variables used in the composite

asr_q06_p	fam_history_5_yes_no	devhx_9_tobacco
asr_q90_p	famhx_4_p	devhx_9_alcohol
asr_q124_p	devhx_8_tobacco	devhx_9_marijuana
asr_q126_p	devhx_8_alcohol	
scrn_hr_smoke	devhx_8_marijuana	

Note. Cells highlighted in blue were collected at both timepoints, cells with no highlighting were only collected at Baseline.

bioRxiv preprint doi: https://doi.org/10.1101/2025.09.01.673428; this version posted September 4, 2025. The copyright holder for this preprint (which was not certified by peer review) is the author/funder, who has granted bioRxiv a license to display the preprint in perpetuity. It is made available under aCC-BY 4.0 International license.

Supplementary Table 2. Model accuracies predicting substance use composites at Baseline broken down by all possible feature combinations

Feature Set	***Intent***	
	Arm 1	Arm 2
All		
PLSR	0.070 (0.49%)	0.083 (0.69%)
RIDGE	0.064 (0.41%)	0.104 (1.08%)
XGB	0.071 (0.50%)	0.088 (0.78%)
Observed Connectivity + Node Embeddings		
PLSR	0.087 (0.75%)	0.089 (0.79%)
RIDGE	0.073 (0.53%)	0.114 (1.30%)
XGB	0.065 (0.43%)	0.104 (1.08%)
Observed Connectivity		
PLSR	0.086 (0.74%)	0.093 (0.87%)
RIDGE	0.072 (0.52%)	0.116 (1.34%)
XGB	0.062 (0.38%)	0.086 (0.84%)
Model Implied Connectivity		
PLSR	0.066 (0.43%)	0.058 (0.33%)
RIDGE	0.058 (0.33%)	0.081 (0.66%)
XGB	0.066 (0.44%)	0.063 (0.40%)
Node Embeddings		
PLSR	0.084 (0.70%)	0.104 (1.08%)
RIDGE	0.070 (0.49%)	0.102 (1.04%)
XGB	0.054 (0.29%)	0.078 (0.61%)
	Access	
	Arm 1	Arm 2
All		
PLSR	0.052 (0.28%)	0.042 (0.18%)
RIDGE	0.038 (0.14%)	0.052 (0.27%)
XGB		0.054 (0.29%)
Observed Connectivity + Node Embeddings		
PLSR	0.061 (0.37%)	0.055 (0.30%)
RIDGE	0.044 (0.19%)	0.068 (0.46%)
XGB		0.078 (0.61%)
Observed Connectivity		
PLSR	0.060 (0.36%)	0.060 (0.36%)
RIDGE	0.043 (0.18%)	0.071 (0.50%)
XGB		0.050 (0.25%)
Model Implied Connectivity		
PLSR	0.024 (0.06%)	0.038 (0.15%)
RIDGE	0.029 (0.08%)	0.050 (0.25%)
XGB		0.032 (0.10%)
Node Embeddings		
PLSR	0.064 (0.41%)	0.044 (0.19%)

bioRxiv preprint doi: https://doi.org/10.1101/2025.09.01.673428; this version posted September 4, 2025. The copyright holder for this preprint (which was not certified by peer review) is the author/funder, who has granted bioRxiv a license to display the preprint in perpetuity. It is made available under aCC-BY 4.0 International license.

RIDGE	0.058 (0.34%)	0.069 (0.48%)
XGB		0.040 (0.16%)
Family-Developmental History		
	Arm 1	Arm 2
All		
PLSR	0.058 (0.33%)	0.052 (0.27%)
RIDGE	0.081 (0.66%)	0.065 (0.42%)
XGB	0.090 (0.81%)	0.112 (1.26%)
Observed Connectivity + Node Embeddings		
PLSR	0.073 (0.54%)	0.059 (0.35%)
RIDGE	0.091 (0.83%)	0.065 (0.42%)
XGB	0.096 (0.92%)	0.089 (0.79%)
Observed Connectivity		
PLSR	0.066 (0.43%)	0.055 (0.30%)
RIDGE	0.086 (0.74%)	0.062 (0.39%)
XGB	0.067 (0.45%)	0.062 (0.39%)
Model Implied Connectivity		
PLSR	0.077 (0.60%)	0.058 (0.33%)
RIDGE	0.076 (0.58%)	0.064 (0.41%)
XGB	0.037 (0.14%)	0.099 (0.98%)
Node Embeddings		
PLSR	0.048 (0.23%)	0.077 (0.60%)
RIDGE	0.081 (0.66%)	0.082 (0.67%)
XGB	0.065 (0.43%)	
Impulsivity (UPPS)		
	Arm 1	Arm 2
All		
PLSR	0.023 (0.05%)	0.043 (0.18%)
RIDGE	0.040 (0.16%)	0.043 (0.18%)
XGB	0.022 (0.05%)	0.002 (0.00%)
Observed Connectivity + Node Embeddings		
PLSR	0.028 (0.08%)	0.046 (0.21%)
RIDGE	0.043 (0.18%)	0.048 (0.23%)
XGB	0.041 (0.17%)	0.014 (0.02%)
Observed Connectivity		
PLSR	0.027 (0.07%)	0.034 (0.12%)
RIDGE	0.042 (0.17%)	0.043 (0.19%)
XGB	0.029 (0.08%)	0.009 (0.00%)
Model Implied Connectivity		
PLSR	0.027 (0.07%)	0.024 (0.06%)
RIDGE	0.044 (0.20%)	0.036 (0.13%)
XGB	0.041 (0.17%)	-0.009 (0.00%)
Node Embeddings		
PLSR	0.034 (0.11%)	0.039 (0.15%)

bioRxiv preprint doi: https://doi.org/10.1101/2025.09.01.673428; this version posted September 4, 2025. The copyright holder for this preprint (which was not certified by peer review) is the author/funder, who has granted bioRxiv a license to display the preprint in perpetuity. It is made available under aCC-BY 4.0 International license.

RIDGE	0.040 (0.16%)	0.049 (0.24%)
XGB	0.029 (0.08%)	0.019 (0.04%)

Note. ‘Arm’ refers to the arm used for confirmation. ‘XGB’, ‘RDIGE’, and ‘PLSR’ refer to models trained and tested with XGBoost, ridge regression, and partial least squares regression respectively. Entries reflect Pearson correlation’s between fitted and observed values during model testing; parentheticals reflect the square of such values on a percent scale, indicating the proportion of variance explained. Results with the node embedding feature set are copied from the main text here to ease comparison.

bioRxiv preprint doi: https://doi.org/10.1101/2025.09.01.673428; this version posted September 4, 2025. The copyright holder for this preprint (which was not certified by peer review) is the author/funder, who has granted bioRxiv a license to display the preprint in perpetuity. It is made available under aCC-BY 4.0 International license.

Supplementary Table 3. Model accuracies predicting substance use composites at the 2 Year Follow-Up broken down by all possible feature combinations

Intent		
	Arm 1	Arm 2
All		
PLSR	0.083 (0.69%)	0.104 (1.09%)
RIDGE	0.082 (0.67%)	0.113 (1.28%)
XGB	0.134 (1.79%)	0.190 (3.61%)
Observed Connectivity + Node Embeddings		
PLSR	0.079 (0.63%)	0.100 (1.00%)
RIDGE	0.079 (0.63%)	0.114 (1.30%)
XGB	0.141 (1.99%)	0.166 (2.77%)
Observed Connectivity		
PLSR	0.077 (0.59%)	0.095 (0.90%)
RIDGE	0.079 (0.63%)	0.115 (1.32%)
XGB	0.145 (2.12%)	0.161 (2.59%)
Model Implied Connectivity		
PLSR	0.088 (0.78%)	0.107 (1.14%)
RIDGE	0.089 (0.81%)	0.110 (1.21%)
XGB	0.179 (3.21%)	0.190 (3.61%)
Node Embeddings		
PLSR	0.099 (0.97%)	0.103 (1.07%)
RIDGE	0.095 (0.91%)	0.115 (1.33%)
XGB	0.184 (3.39%)	0.194 (3.76%)
Access		
	Arm 1	Arm 2
All		
PLSR	0.242 (5.85%)	0.186 (3.48%)
RIDGE	0.136 (1.85%)	0.120 (1.45%)
XGB	0.368 (13.54%)	0.364 (13.25%)
Observed Connectivity + Node Embeddings		
PLSR	0.195 (3.79%)	0.202 (4.08%)
RIDGE	0.142 (2.02%)	0.135 (1.84%)
XGB	0.355 (12.60%)	0.377 (14.21%)
Observed Connectivity		
PLSR	0.244 (5.94%)	0.198 (3.93%)
RIDGE	0.134 (1.78%)	0.134 (1.78%)
XGB	0.372 (13.84%)	0.363 (13.18%)
Model Implied Connectivity		
PLSR	0.190 (3.61%)	0.222 (4.94%)
RIDGE	0.127 (1.62%)	0.112 (1.26%)
XGB	0.381 (14.52%)	0.367 (13.47%)
Node Embeddings		
PLSR	0.244 (5.96%)	0.160 (2.56%)

bioRxiv preprint doi: https://doi.org/10.1101/2025.09.01.673428; this version posted September 4, 2025. The copyright holder for this preprint (which was not certified by peer review) is the author/funder, who has granted bioRxiv a license to display the preprint in perpetuity. It is made available under aCC-BY 4.0 International license.

RIDGE	0.154 (2.04%)	0.143 (2.04%)
XGB	0.391 (15.29%)	0.377 (14.21%)
Family-Developmental History		
	Arm 1	Arm 2
All		
PLSR	0.359 (12.90%)	0.394 (15.51%)
RIDGE	0.382 (14.57%)	0.431 (18.54%)
XGB	0.445 (19.78%)	0.580 (33.59%)
Observed Connectivity + Node Embeddings		
PLSR	0.284 (8.08%)	0.339 (11.48%)
RIDGE	0.387 (15.01%)	0.439 (19.31%)
XGB	0.474 (22.43%)	0.531 (28.15%)
Observed Connectivity		
PLSR	0.320 (10.23%)	0.352 (12.40%)
RIDGE	0.393 (15.44%)	0.445 (19.84%)
XGB	0.475 (22.56%)	0.555 (30.84%)
Model Implied Connectivity		
PLSR	0.386 (14.93%)	0.456 (20.76%)
RIDGE	0.396 (15.68%)	0.451 (20.33%)
XGB	0.479 (22.94%)	0.544 (29.62%)
Node Embeddings		
PLSR	0.311 (9.65%)	0.379 (14.34%)
RIDGE	0.446 (19.89%)	0.516 (26.58%)
XGB	0.504 (25.40%)	0.543 (29.49%)
Impulsivity (UPPS)		
	Arm 1	Arm 2
All		
PLSR	0.020 (0.04%)	0.050 (0.25%)
RIDGE	0.014 (0.02%)	0.020 (0.00%)
XGB	0.011 (0.01%)	0.054 (0.29%)
Observed Connectivity + Node Embeddings		
PLSR	0.028 (0.08%)	0.063 (0.40%)
RIDGE	0.014 (0.02%)	0.031 (0.10%)
XGB	0.024 (0.06%)	0.065 (0.42%)
Observed Connectivity		
PLSR	0.031 (0.09%)	0.052 (0.26%)
RIDGE	0.014 (0.02%)	0.030 (0.10%)
XGB	0.015 (0.02%)	0.068 (0.45%)
Model Implied Connectivity		
PLSR	0.017 (0.03%)	0.028 (0.08%)
RIDGE	0.003 (0.00%)	0.014 (0.02%)
XGB	-0.010 (0.01%)	0.020 (0.04%)
Node Embeddings		
PLSR	0.051 (0.26%)	0.089 (0.79%)

bioRxiv preprint doi: https://doi.org/10.1101/2025.09.01.673428; this version posted September 4, 2025. The copyright holder for this preprint (which was not certified by peer review) is the author/funder, who has granted bioRxiv a license to display the preprint in perpetuity. It is made available under aCC-BY 4.0 International license.

RIDGE	0.029 (0.08%)	0.031 (0.10%)
XGB	-0.00 (0.00%)	0.089 (0.79%)

Note. ‘Arm’ refers to the arm used for confirmation. ‘XGB’, ‘RDIGE’, and ‘PLSR’ refer to models trained and tested with XGBoost, ridge regression, and partial least squares regression respectively. Entries reflect Pearson correlation’s between fitted and observed values during model testing; parentheticals reflect the square of such values on a percent scale, indicating the proportion of variance explained. Results with the node embedding feature set are copied from the main text here to ease comparison.
